# Urban climate–NCD syndemics in LMICs: a transdisciplinary framework for Health action

**DOI:** 10.1080/16549716.2026.2650971

**Published:** 2026-04-22

**Authors:** Meelan Thondoo, Feyisayo Wayas, Lidia Morais, Elis Borde, Charles Obonyo, Mikateko Mafuyeka, Damilola Odekunle, Victor Onifade, Tiago Canelas, Motlatso Godongwana, Lambed Tatah, Clarisse Mapa-Tassou, Edwin Ngwa, Awah Tchouaffi, Felix Assah, Opeyemi Babajide, Grace Githiri, Georgiana Gordon-Strachan, Taibat Lawanson, Tolu Oni

**Affiliations:** a MRC Epidemiology Unit, University of Cambridge, Cambridge, UK; b Research Centre for Health Through Physical Activity, Lifestyle & Sport, Division of Physiological Sciences Department of Human Biology, Faculty of Health Sciences, University of Cape Town, Cape Town, South Africa; c Observatory for Urban Health in Belo Horizonte, Federal University of Minas Gerais (OSUBH/UFMG), Belo Horizonte, Brazil; d Centre for Global Health Research, Kenya Medical Research Institute, Kisumu, Kenya; e The SAMRC/Wits Centre for Health Economics and Decision Science (PRICELESS SA), School of Public Health, University of the Witwatersrand, Johannesburg, South Africa; f Centre for Housing and Sustainable Development, Department of Urban and Regional Planning, University of Lagos, Cape Town, South Africa; g Developmental Pathways for Health Research Unit, University of the Witwatersrand, Johannesburg, South Africa; h Health of Population in Transition, Research Group, The University of Yaoundé I, Yaoundé, Cameroon; i Urban Health Collaborative, Drexel University, Philadelphia, USA; j Policy, Legislation and Governance Section, the Urban Practices Branch (UPB) of the Global Solutions Division at UN-Habitat Headquarters United Nations Habitat, Nairobi, Kenya; k Tropical Metabolism Research Unit, Caribbean Institute for Health Research, University of West Indies, Kingston, Jamaica

**Keywords:** Transdisciplinary research, clustering health impacts, climate change, non-communicable diseases, Global South

## Abstract

**Background:**

Climate change poses a major threat to global health. Its synergistic interaction with non-communicable diseases (NCDs), responsible for 74% of global deaths, demands urgent, integrated action.

**Objectives:**

This study critically examines how transdisciplinary approaches can uncover and address structural determinants underpinning climate change–NCD syndemic hazards, while accounting for power dynamics through meaningful stakeholder engagement in urban contexts.action.

**Methods:**

A mixed-methods design combined quantitative data (syndemic variable counts, stakeholder categories, pre- and post-workshop surveys) with qualitative inductive analysis of stakeholder narratives to identify themes on health impacts of syndemic factors, climate events, and adaptive strategies across seven cities in South Africa, Nigeria, Jamaica, Brazil, Kenya, and Cameroon. Syndemic relationships were analyzed through five critical lenses: structural determinants; climate–health linkages as manifestations of structural vulnerability; stakeholder power dynamics; transformative community engagement; and critical reflection on transdisciplinary processes.

**Results:**

A total of 172 diverse stakeholders prioritized 24 predefined syndemic variables across three domains – climate and natural hazards, built environment and physical activity, and food environment – and contributed 71 additional context-specific variables. Validated relevant crosscutting variables included healthy food outlets, walkability, sidewalk presence, and air pollution. Stakeholders emphasized the need for comprehensive, context-specific strategies and inclusive community engagement to address the broad health consequences of climate events.

**Conclusion:**

Transdisciplinary approaches enable deeper understanding of syndemic relationships by fostering counter-hegemonic knowledge production. We propose an Integrated Syndemic Climate–Health Framework to refine transdisciplinary processes for enhanced research, localized interventions, and iterative evaluation in Low- and middle-income countries.

## Paper Context

- **Main findings:** Transdisciplinary approaches can enable a better understanding of syndemic climate–health relationship, especially in low- and middle-income settings.
- **Added knowledge:** The authors proposed a novel Integrated Syndemic Climate–Health Framework to examine specific health effects of climate events.
- **Global health impact for policy and action:** This study contributes to critical transdisciplinary approach research in low- and middle-income countries to reduce climate–health inequities and relevant public health.

## Background

Climate change and non-communicable diseases (NCDs) represent intersecting global health crises whose impacts are profoundly shaped by structural inequities within a globalized world. The convergence of these multiple health threats, conceptualized as a ‘syndemic’, manifests with particular intensity in urban settings of low- and middle-income countries (LMICs) [1–3] where 80% of the global urban population is projected to live by 2050 [4]. In these rapidly urbanizing contexts, the climate–NCD syndemic is not merely a coincidence of environmental and health challenges, but rather a manifestation of systematic power imbalances that disproportionately expose marginalized communities to multiple, interacting health risks.

The structural determinants underlying these syndemic relationships can be traced to global economic systems that perpetuate environmental exploitation and health inequities. Deaths due to NCDs in LMICs are expected to increase from 30.8 million per year in 2015 to 41.8 million by 2030 [5], a trajectory driven not only by changing lifestyles but by the unequal distribution of resources, opportunities, and exposures within the global political economy. Insufficient physical activity (PA) and diet-related metabolic risk factors, including body mass index, fasting glucose, and systolic blood pressure [6], reflect not just individual behaviours but systematically unequal access to healthy environments shaped by globalization processes.

Transdisciplinary (TD) research offers potentially transformative approaches to both understanding and challenging these complex power dynamics. TD is defined as ‘a reflexive, integrative, method-driven scientific principle aiming at the solution or transition of societal problems and concurrently of related scientific problems, by differentiating and integrating knowledge from various scientific and societal bodies of knowledge’ [7]. However, critical analyses have identified that many TD literature emphasizes theoretical potential rather than practical application [8] particularly in addressing structural determinants of health. For example, TD studies in environmental governance and health systems research have often focused on conceptual integration and knowledge frameworks rather than the concrete operational mechanisms needed for implementation, such as decision-making protocols, cross-sector coordination, or evaluation processes [8–10]. These works demonstrate the intellectual strength of TD theory but reveal a persistent gap in translating these principles into applied, context-sensitive practices. Our study responds to this gap by operationalising TD principles through participatory co-design and iterative evaluation, showing how TD approaches can be practically implemented in climate–health research. This gap is especially pronounced in LMICs [9], with even fewer TD studies critically examining the power relationships underlying climate change-health linkages [10].

Using our novel TD research project focused on exploring population exposure to syndemic hazards and approaches to mitigating diet and PA impacts of these exposures, we critically examine how transdisciplinary approaches can reveal and address the structural determinants underlying climate change-NCD syndemic hazards, while challenging existing power dynamics through meaningful stakeholder engagement in urban contexts. We interrogate how globalizing forces shape these syndemic relationships and explore transformative, equity-centered solutions grounded in a TD approach. We follow five key lines of critical inquiry which also constitutes our study’s five objectives.

1. Analyse syndemic relationships through a structural determinants lens that integrate linkages between climate change, diet and PA environments and health.
2. Assess climate–health linkages as manifestations of structural vulnerability examining how health impacts of climate events reflect and reinforce existing health inequities.
3. Identify power dynamics among local actors/stakeholders to engage meaningfully with those involved in responses while examining whose voices are privileged or marginalized.
4. Explore transformative community engagement strategies to challenge dominant knowledge systems and create spaces for counter-hegemonic approaches.
5. Critically reflect on TD processes for transformation to challenge conventional approaches and develop more equitable methodologies based on lessons learned.

Our analysis draws on stakeholder engagement workshops conducted through the Global Diet and Physical Activity Research (GDAR) Network’s research programme across diverse urban contexts including South Africa (Johannesburg and Cape Town), Nigeria (Lagos), Jamaica (Kingston), Brazil (Belo Horizonte), Kenya (Kisumu), and Cameroon (Yaoundé) [11]. In these settings, built and food environments reflect complex power relationships across public and private sectors and civil society, shaped by global economic forces and local realities. The GDAR Network has established transdisciplinary partnerships that explicitly acknowledge power dynamics across diverse academic disciplines and societal actors to co-design and co-produce research addressing upstream structural determinants of diet and PA behaviors in the context of socio-economic inequality [11]. This study represents a significant contribution to critical transdisciplinary research in LMICs by demonstrating how such approaches can not only generate integrated solutions but challenge the power structures that underlie health inequities in a globalized world.

### Theoretical frameworks underpinning the climate–NCD syndemic

Current theoretical frameworks addressing climate–health relationships have evolved substantially but continue to face challenges in fully transcending disciplinary boundaries and addressing power dynamics that shape both knowledge production and health outcomes. For instance, the Planetary Health framework, pioneered by the Rockefeller Foundation-Lancet Commission, conceptualizes human health as fundamentally dependent on Earth’s natural systems, emphasizing how disruptions to these systems impact human wellbeing [12]. While comprehensive in linking environmental degradation to health outcomes at macro-level, this framework has been critiqued for providing limited guidance for operationalizing transdisciplinary collaboration locally, inadequately addressing urban contexts where multiple exposures concentrate, and paying insufficient attention to power differentials in knowledge production and governance responses [13].

Similarly, the One Health approach [14] emphasizes interconnections between human, animal, and environmental health and maintains integrated ecological and health systems thinking but has been critiqued for limited engagement with social determinants and structural power dynamics [15]. Of relevance are also the Ecosocial theory and the Syndemic theory. The Ecosocial Theory framework developed by Krieger [16] offers important insights on embodiment, accountability, and agency but lacks specific guidance for climate–NCD interactions in urban contexts. The Syndemic theory, applied to climate change and health by Singer et al. [17], emphasizes how biological and social interactions amplify disease burden within populations experiencing structural violence. However, as Mendenhall et al. [2] observe, syndemic applications often remain focused on individual-level disease interactions rather than broader political-economic structures, with limited practical guidance for transdisciplinary research and intervention design.

These climate–health frameworks largely operate in parallel to, rather than integration with, transdisciplinary research approaches. Current transdisciplinary frameworks face their own critical limitations. First, they often overlook the complex interplay of multiple syndemic hazards in urban contexts [18,19], missing the intricate relationships between air pollution, urban density, and food security. Second, most TD approaches address broader environmental impacts but fail to delve into the compounded effects of climate and health hazards in urban settings [13], leaving a gap in understanding these interconnected factors. In practice, these frameworks tend to analyse singular exposures, such as air pollution, flooding, or heat-related illness, without considering how these interact with social and infrastructural determinants like food insecurity, housing conditions, and mobility constraints to exacerbate noncommunicable disease risks. For example, recurrent flooding can simultaneously disrupt food systems, heighten exposure to vector-borne diseases, and intensify psychosocial stress, yet such synergistic pathways remain poorly captured in existing models. Lastly, although integrating policy frameworks with environmental health strategies lays a foundation for interventions [9,20], literature often neglects the complexity of stakeholder interactions and the practicalities of implementing transdisciplinary approaches in climate–health contexts. The absence of frameworks that effectively bridge climate–health understanding with practical transdisciplinary implementation represents a significant gap, particularly for addressing urban syndemic challenges in LMIC settings.

## Methods

### Study design

We employed a mixed-methods approach encompassing quantitative and qualitative data collection and data analysis of stakeholder workshops conducted in Johannesburg, Cape Town, Lagos, Kingston, Belo Horizonte, Kenya, and Yaoundé. The quantitative data included the number and categories of stakeholders, frequency counts of climate syndemic variables, and results from pre- and post-workshop surveys, while the qualitative data comprised transcripts, researcher notes, plenary discussions, and outputs from co-creation tools such as whiteboards, sticky notes, and scorecards. Mixed-methods approach was utilised for a nuanced interpretation of the findings. Workshops were full-day, in-person events conducted in stakeholders’ preferred languages (i.e. English or their local languages) and adjusted to each site’s specific climate hazards and audiences. Detailed description of the stakeholder workshops is shown in Supplementary file 1. This study adhered to the Good Reporting of a Mixed Methods Study (GRAMMS) checklist (Checklist file 1).

#### Data collection

We conducted seven stakeholder engagement workshops between June 2022 and March 2023. Workshop activities were highly interactive, utilizing exercises, case study reviews, plenary discussions, and pre- and post-workshop surveys. A workshop facilitator’s guide was developed to ensure harmonisation across sites (Supplementary file 2). Data were captured through standardized reporting templates, real-time anonymous surveys (e.g. mentimeter), co-creation tools (e.g. whiteboards, sticky notes, scorecards), and researcher notes. In Johannesburg, Cape Town, and Belo Horizonte, workshop sessions were partially audio-recorded.

#### Data analysis

We conducted analysis between March and November 2023. A core research group across four sites analysed the workshop data using Microsoft Excel for quantitative descriptive analysis and NVivo software for qualitative content analysis. The inductive content analysis served to identify themes across sites, assessing the impact of syndemic variables and climate events on health, and analysis of adaptive strategies by policy, commercial, and community actors. A data extraction sheet (Supplementary file 3) was used to organize the themes based on predefined inquiry lines developed through collaborative sessions among researchers. When unclear data or discrepancies in coding arose, local teams provided clarification, and consensus was reached among the core research group before data synthesis and feedback from the broader GDAR network.

#### Ethical considerations

The data collection method posed minimal risk to participants. Ethical approval was granted by The Cambridge Psychology Research Ethics Committee (Application No: PRE.2022.074), as well as local ethics committees in each city. Informed consent was obtained from all participants. Discussions followed Chatham House rules, and all data were anonymized by replacing participant names with identification numbers. Ethical principles, including autonomy, confidentiality, justice, and beneficence, were strictly followed as outlined in the Declaration of Helsinki.

### Stakeholder engagement workshops

#### Framework

**Figure 1.**
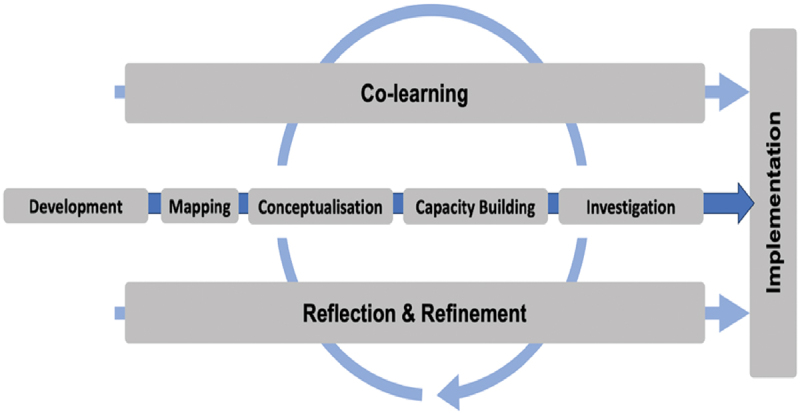
Eight-phase transdisciplinary framework adapted from Pineo et al. [19].

The workshops follow an eight-phase framework (Figure 1) adapted from Pineo et al.‘s transdisciplinary research model [19].

To pre-develop and conceptualize the stakeholder engagement workshops, the research team held several co-creation meetings. This was followed by a capacity building and training programmes for researchers and facilitators to ensure harmonization of ethical protocols and workshop design.

#### Workshop objectives

The three workshop objectives were (1) to engage relevant stakeholders (e.g. identify their priorities and incorporate their perspectives) to co-create the research agenda and refine study objectives; (2) to examine and apply different theoretical and practical participatory TD methodologies and tools for improving stakeholder engagement, focusing on urban actors and policies and (3) to utilize an integrated knowledge translation process for research findings to inform urban health policies, interventions and development strategies.

### Participant selection and attendance

The research team mapped stakeholders to create a master list of climate, food, and built environment stakeholders in each site. Stakeholder mapping using the Biodiversa toolkit (www.Biodiversa.org) was used to categorize participants by power, influence, and relevance across six typologies (e.g. policy, commercial, community, academia, technicians, not-for-profit). Contact details were gathered, and contacted stakeholders suggested additional relevant contacts. An initial masterlist of 410 stakeholders were invited to the workshop.

### Workshop activities

**Figure 2.**
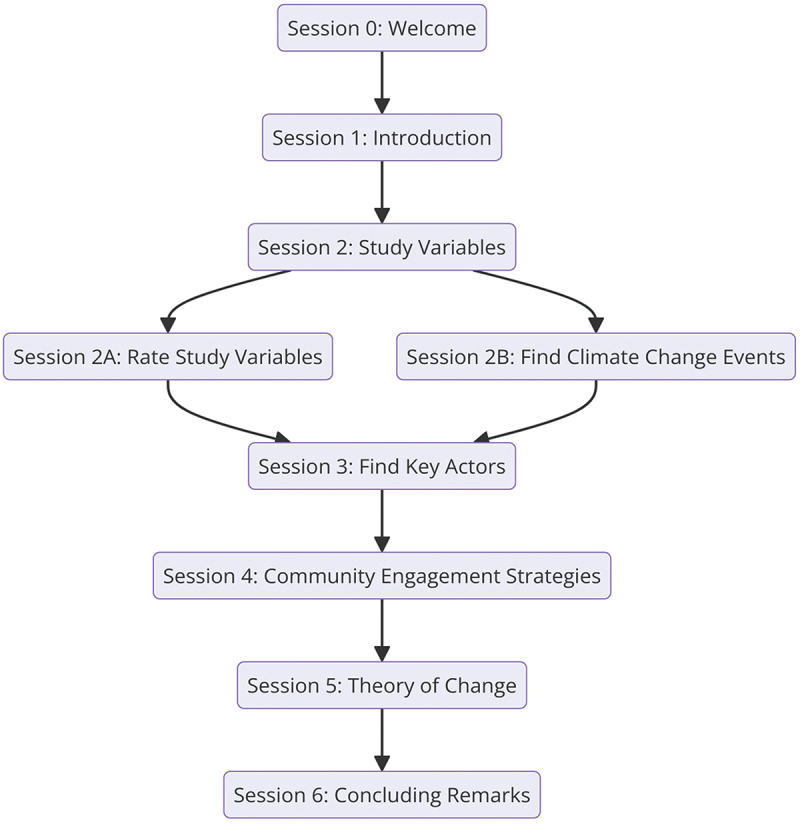
Workshop activities.

The workshops consisted of six main activities (sessions 2–5) aligned with this paper’s five key lines of inquiry which are also the study’s objectives (Figure 2). The workshop consisted of a mix of plenary and breakout sessions. Participants were presented with 24 predefined syndemic variables across three domains: climate and natural hazards, built environment related to physical activity, and food environment based on literature and pre-workshop researchers’ discussions in a standardized template.

To validate predefined climatic variables based on their relevance, importance, and associated NCD-related health impacts, stakeholders worked in groups to review each variable collectively. Each group colour-coded the variables as *relevant* (green), *not relevant* (red), or *partially relevant* (amber/yellow) to indicate mixed opinions. A variable was considered validated for high relevance when the majority (over 70%) of participants in a group marked it as green, split validation when responses were mixed (green and red in roughly equal proportions), and validated as low relevance when most participants marked it red. Participants also ranked each validated variable on a scale of 1–5, with 5 denoting highest importance, and identified corresponding stakeholders, engagement mechanisms, and data sources. They further discussed additional syndemic hazards to inform environmental audits, listed major climate events from the past decade and their health impacts, and identified relevant data-holding institutions.

Subsequent activities focused on identifying key local actors across critical sectors to better understand how community, policy, and commercial actors (and their respective institutions/groups) respond, cope, and adapt to climate change shocks. The participants then focused on identifying community engagement and involvement (CEI) strategies as a core component of the research and the importance of undertaking the research in collaboration with the groups most likely affected by the research outcomes. They were finally invited to instigate discussion and feedback on the existing approach to the research proposal’s Theory of Change (ToC) to address the critical underlying assumptions expected to be met to bring about change at the societal level.

To identify stakeholders willing to continue engagement after the workshops, a post-workshop survey was administered at the conclusion of each session to collect contact details and determine participants’ desired levels of engagement.

## Results

Our research interrogates how transdisciplinary approaches can disrupt and transform existing power structures that perpetuate climate–NCD syndemics.

### Stakeholders workshop attendance

A total of 172 stakeholders (42%) were selected by convenience sampling and participated in seven workshops in seven cities. They comprised policymakers (34%), academic actors and technicians (27%), community actors (15%), not-for-profit actors (16%), and commercial actors (8%) (Table 1). The initial masterlist contained 410 invited stakeholders, indicating an overall attendance rate of 42%, with ‘academia and technicians’ and ‘not-for-profit’ actors showing the highest (69%) and lowest (22%) attendance rates, respectively.

**Table 1.** Stakeholder invitations and attendance by type across sites.

	Commercial	Community	Not-for-profit	Policy	Academia; Technicians	Grand total
Invited from master list stakeholder mapping (N)	63	45	122	113	67	410
Attended (N)	14	26	27	59	46	172
Attendance rate (%)	22	58	22	52	69	42

Stakeholders’ attendance across study sites varied significantly. For instance, there was low attendance of community actors in Kingston, Kisumu and Johannesburg and no attendance of commercial actors in Belo Horizonte and Yaoundé. Not-for-profit actors and policy actors were present across all sites. After each workshop, stakeholders received a report as a dissemination tool (Supplementary file 4) and means to continue engagement with the research and facilitate collaboration across actors. Following our five lines of critical inquiry (objectives), we examine: (1) syndemic relationships through a structural determinants lens; (2) power dynamics in climate–health linkages; (3) stakeholder representation and marginalization; (4) transformative community engagement strategies; and (5) critical reflection on TD processes as tools for challenging conventional approaches.

### Syndemic relationships through a structural determinants lens

Across the seven workshops, stakeholders assessed and colour-coded the 24 predefined syndemic variables to indicate levels of consensus on their relevance to climate–NCD linkages. Variables marked in green reflected those validated as relevant by most participants (strong consensus), yellow indicated mixed or split validation across groups, red denoted variables deemed less relevant by most (Supplementary file 5).

High consensus of relevance (green) emerged for variables such as *healthy food outlets*, *walkability*, *sidewalk presence*, and *air pollution (PM_2.5_ and PM_10_)*, which participants consistently associated with urban health and environmental quality. By contrast, variables like *sidewalk obstruction*, *number of public transport points*, and *retail establishments* received limited support (red), indicating lower perceived relevance or weaker policy linkages. Split validation (yellow) occurred for variables whose importance varied by local context, for example, *street lighting*, *green space presence*, and *road safety* were considered crucial in some sites but peripheral in others.

This validation process also revealed cross-site variation in structural priorities. The built environment domain showed the greatest divergence: stakeholders in Lagos and Yaoundé viewed *number of shops* and *public transport points* as less critical compared to Johannesburg, where these were seen as indicators of accessibility and safety. Such variation reflects differences in urban form, infrastructure quality, and governance structures between cities.

Following this exercise, stakeholders proposed an additional 71 context-specific variables, particularly within the food environment domain, highlighting systemic factors such as *food production chains*, *corporate food systems*, *inflation of food products*, and *overregulation*, as well as social determinants like *gendered mobility constraints* and *household food expenditure share* (Supplementary file 6). These additions emphasised that existing frameworks often underrepresent structural inequities and community-level dynamics shaping the climate–NCD syndemic.

### Climate–health linkages as manifestations of structural vulnerability

Stakeholder deliberations revealed complex and interconnected health impacts resulting from climate events (Table 2, Supplementary file 7). Direct impacts included fatalities from floods, landslides, and extreme heat, while indirect impacts manifested through multiple pathways, reflecting and reinforcing existing health inequities. Salient examples included mental health issues arising from psychosocial stress and a diminished quality of life and waterborne diseases, exacerbated by contaminated water and shortages, leading to diarrhoea and dehydration.

**Table 2.** Condensed linkages between climate events and health reported by participants (see full list in Supplementary 3).

Category	Representative health impacts
Deaths	Floods, landslides, heat-related mortality, loss of animal lives
Mental Health	Psychosocial distress, sadness, disruption of daily life
Waterborne Diseases	Leptospirosis, malaria, dengue, diarrhea from poor water quality
Food Environment	Food insecurity, malnutrition, supply chain disruption, loss of aquatic resources
Pollution	Air and water contamination, smoke from waste burning, wildfire-related air pollution
NCD Risk Factors	Heat stroke, respiratory illness, dehydration, waste-burn emissions
Physical Activity	Reduced outdoor activity, damaged infrastructure, unsafe environments
Other	Poor indoor ventilation, reduced sanitation, disrupted breastfeeding routines

Food security was identified as severely compromised through multiple mechanisms tied to structural vulnerability: land degradation, disrupted food supply chains, contamination, power outages affecting storage, and infrastructure damage to food retail establishments. These disruptions resulted in increased food prices, reduced food quality, and malnutrition, disproportionately affecting communities with less resilient infrastructure and fewer resources. In some cases, stakeholders reported that these impacts even led to forced migration.

Climate impacts on physical activity infrastructure revealed how climate events diminish opportunities for healthy behaviors, particularly affecting those reliant on public spaces. Stakeholders identified that damaged walkways, air pollution, and compromised sports facilities had more severe impacts in communities already facing limited options for physical activity. Pollution from vehicles, waste, and wildfires degraded air and water quality, causing respiratory issues and a decline in public health. Furthermore, NCDs like cardiac- and cancer-related illnesses were understood to emerge from environmental stressors, while reduced physical activity due to damaged infrastructure and pollution further impacts well-being and exercise opportunities.

The resulting impacts create cascading effects on health systems, increasing pressure on hospitals and reducing sanitation. Overall, participants in each site reported that these interconnected factors highlight the urgent need for comprehensive strategies to address the broad spectrum of health consequences in the aftermath of climate events. These findings demonstrate that health impacts of climate events are not randomly distributed but follow existing patterns of disadvantage, reinforcing structural vulnerabilities in marginalized communities. The transdisciplinary approach allowed stakeholders to articulate these structural vulnerabilities in ways that transcended siloed understandings of climate impacts.

### Power dynamics among local actors/stakeholders

The comprehensive actor mapping across seven cities (Supplementary file 8) revealed which entities are recognized as influential in climate response and whose voices are privileged or marginalized in addressing climate–NCD syndemic hazards. Government agencies dominated the response landscape across all sites, indicating their outsized power in setting policy agendas. For example, in Lagos, these included government agencies such as the Lagos Resilience Office (LASRO), the Ministry of Environment, and the Ministry of Physical Planning and Urban Development, which play pivotal roles in policy making and infrastructure development.

Civil society organizations and social movements, including community development associations (CDAs) and NGOs, were perceived as vital for grassroots engagement and advocacy but were often positioned as implementers rather than agenda-setters. Academic institutions, such as universities and research bodies, were seen to contribute essential research and innovation, yet their influence varied considerably across sites. Industry actors, including telecommunication companies like MTN and Airtel, and corporations like Nestlé and Access Bank, were reported as actors that could provide financial and logistical support, revealing the complex role of commercial interests in climate response.

Environmental and health-focused organizations, including the World Health Organization (WHO) and various local health educators, were noted as offering expertise in disaster risk management and community health, while media outlets, such as Enviro News, were deemed crucial for communication and public awareness. The inclusion of informal recyclers and local businesses, such as market vendors and farmers, highlighted grassroots actors whose voices are often marginalized despite their direct experience with climate impacts. This comprehensive mapping of actors revealed significant power imbalances in who shapes climate–health responses.

The uneven representation of different stakeholder categories in the workshops themselves (Table 1) reflected these existing power imbalances. Commercial sector representatives and policymakers were notably underrepresented at several sites, demonstrating the challenges in engaging powerful actors in transdisciplinary processes. Participants noted that the transdisciplinary approach brought together actors who ‘would normally not be in the same room,’ highlighting how this methodology challenged existing siloed power structures by creating unprecedented dialogue opportunities. Stakeholders observed a ‘disconnection between policy actors and implementation actors’ during the Investigation Phase (Figure 1), revealing power dynamics that affect implementation. In Lagos and Johannesburg, workshops created contested political spaces where community members directly confronted policymakers about implementation gaps and structural inequities perpetuated through climate policies that exacerbate socioeconomic vulnerabilities.

Significantly, participants identified the problematic role of certain corporate actors (such as ‘Big Oil’ in South Africa) alongside potential partners like telecommunication companies – highlighting tensions between commercial interests and climate–health equity. The identification of informal recyclers and social movements demonstrates how marginalized actors seek recognition despite systematic exclusion from formal response structures.

The transdisciplinary approach made these power dynamics visible and created opportunities to engage more meaningfully with those typically marginalized in climate–health decision-making. However, challenges in engaging certain sectors, particularly commercial actors and policymakers, were documented across sites, reflecting entrenched power dynamics that limit multisectoral collaboration. These findings suggest that effective climate response requires not only identifying diverse actors but transforming the power relationships between them, particularly addressing how global economic interests shape local response capacities.

### Transformative community engagement strategies

Stakeholders proposed several strategies to strengthen community engagement and challenge knowledge and impact around climate–health solutions (Table 3). A consistent recommendation across sites was the need to develop strategic pathways toward behaviour change among different actors, emphasizing the importance of identifying underlying conditions necessary for planned change to occur.

**Table 3.** Transformative community engagement initiative strategies.

Community Engagement Initiatives (CEI)	Strengthen the Network through shared learning and knowledge exchange	Bridging the gap between policy and civil society actors	Raise the profile of the work of the Network to improve reach with academic and funding actors
Stakeholder engagement (face-to-face/real time)		x	
Increase visibility on social media during key days and events			x
Develop and disseminate a newsletter.	x	x	
Disseminate findings at academic events.			x
Disseminate findings at policy fora.		x	
Use the Network website to profile country members, activities, and outputs.	x		x
Evaluate and tailor CEI strategy.	x	x	x
Explore/embrace creative participatory outputs.	x	x	x

They reported the need to act on a strategic pathway towards behaviour change from different actors, build better linkages between research findings and policy change, and encourage the involvement of different income groups in the co-design phase of the research. There was consensus across sites that it is important to better identify the underlying conditions and resources that need to exist for planned change to occur. For instance, a stakeholder in Johannesburg pointed out that identifying what type of behaviour change was required from different actors is essential for the research to have a higher impact. Similarly, a participant from Cape Town called for a ‘more strategic approach to climate activism,’ which can be achieved by ‘involving intended communities upfront and identifying what type of behaviour can impede the project’s success.’ It was also a common agreement across sites that the link between the implementation of research findings and policy change (particularly regarding climate change) needs to be better strengthened in the ToC by ‘getting political buy-in upfront and involving different income groups in the co-design phases.’ Equally, across sites, there was a call for promoting awareness of specific measures for NCD prevention and the importance of ‘regular feedback loops with stakeholders.’

To enhance impact, stakeholders recommended three key community engagement and impact (CEI) strategies that create spaces for counter-hegemonic approaches: strengthening knowledge exchange within the research network, bridging gaps with policy and civil society actors, and raising the profile of transdisciplinary health research (Table 3). Stakeholders highlighted the importance of strengthening the GDAR network through shared learning and knowledge exchange, utilizing online platforms to promote events, webinars, and research opportunities, thereby elevating the profile of Early Career Researchers (ECRs). This included organizing annual meetings, ECR forums, mentoring sessions, and engagement with the Network Steering Group (NSG). They emphasized the need to bridge the gap with policy and civil society actors by understanding their perspectives, fostering trust, and ensuring meaningful community involvement in research design and dissemination of reports including actor insights across commercial, policy and community domains. They underlined the role of the Network to advocate for transdisciplinary health research on urbanization and climate change among academic circles and donors.

The recommendation for ‘regular feedback loops with stakeholders’ suggests a more democratic and inclusive approach to knowledge creation that values diverse perspectives. Equally, the suggestion for ‘creative participatory outputs’ as a cross-cutting strategy offers innovative communication approaches that can reach beyond academic audiences and challenge traditional knowledge dissemination methods by centering community perspectives alongside technical expertise.

### Critical reflections on TD processes for transformation

The transdisciplinary approach embedded in the eight-phase workshop design (Figure 1) served as a valuable co-production platform for fostering collaboration, knowledge sharing, and strategic planning among actors who ‘would normally not be in the same room’ (Participant, Yaoundé, Cameroon workshop) and offered insights into developing more equitable approaches to address the climate–NCD syndemic (Supplementary file 9). Our reflection process revealed several significant methodological challenges. We found that knowledge integration was hindered by participants using different terminologies and ‘understanding of concepts’ across disciplines, highlighting fundamental epistemological barriers in transdisciplinary work. Additionally, despite being in the same room, participants ‘often exchanged in silos,’ revealing how deep-seated disciplinary and sectoral boundaries persist even in carefully designed collaborative spaces. The process also underscored how power hierarchies and trust deficits between different stakeholder groups created barriers to effective knowledge co-production, with participants requesting for ‘creating intentional spaces to mix and remix’ that would help them create more rapport and exchange from equal standpoints (Supplementary file 9). Reflections on building trust and addressing power hierarchies provided important lessons for more equitable transdisciplinary processes. Participants across sites articulated how extractive research relationships can reproduce existing inequities and suggested approaches to transform these dynamics through more democratic engagement processes such as ‘open communication’ and ‘alignment of expectations among stakeholders’. Examples for iterative learning and cross-observations are provided for each phase in supplementary file 9, where critical engagement extended beyond simply documenting diverse perspectives to actively challenging conventional participation techniques while creating space for alternative ways of understanding and addressing complex problems around meaningful engagement. For instance, during the Co-Learning Phase, challenges with actor representation persisted, with a notable disparity between researchers and non-researchers at workshops. In Yaoundé, we observed that when invitations were extended to institutions, they tended to send representatives based on availability rather than expertise relevance, diminishing meaningful participation. By contrast, when individuals were directly invited based on their experience with climate or NCD issues, engagement quality improved significantly. This reflection led to the recommendation for more targeted invitations to individuals rather than institutions, challenging approaches that often privilege institutional participation over individual engagement.

## Discussion

GDAR, comprising 42 researchers across 9 institutions in 8 cities and 7 countries with more than 30 partners represents a pioneering LMIC-based transdisciplinary initiative [11]. The stakeholder workshops engaged over 170 participants from community, policy, and commercial sectors, creating unprecedented spaces for dialogue. This study advances syndemic research by empirically demonstrating how transdisciplinary methods reveal determinants that remain invisible within conventional approaches [21,22].

By facilitating hands-on exercises, stakeholders identified complex syndemic relationships, with consensus across sites regarding air pollution, urban density, and food availability as priorities (Table 1; Supplementary file 5). Participants articulated 71 additional variables beyond conventional frameworks – particularly in food environments – revealing critical structural factors shaped by global market forces, systematic exclusion of local voices, and class and gender inequities that are typically overlooked [23,24]. In line with our first line of critical inquiry, these findings highlight how structural determinants such as governance, infrastructure, and food systems underpin unequal exposures and shape the architecture of climate–NCD interactions. This interpretation aligns with global literature on commercial and structural determinants of health, reaffirming that syndemics emerge from political–economic systems rather than individual behaviours.

Going beyond studies documenting isolated climate impacts [25], our research illuminates climate–health linkages as manifestations of structural vulnerability (second line of inquiry), showing how cascading impacts from floods, heat, and food insecurity affect both direct and indirect health outcomes, including mental health and nutrition (Supplementary file 7). By integrating diverse stakeholder perspectives, we demonstrate that these effects are not randomly distributed but follow entrenched socio-economic hierarchies in vulnerable urban communities [17,26]. This aligns with theories of structural vulnerability in climate–health research [27,28] which emphasise how cumulative disadvantage shapes adaptive capacity and exposure to climate hazards.

Findings from Supplementary file 5 revealed asymmetrical power dynamics among actors, where state and commercial interests dominated decision-making while community perspectives were under-represented (third line of inquiry). However, through transdisciplinary engagement, workshops created dialogic spaces in which community members discussed structural inequities directly with policymakers, fostering opportunities for more democratic knowledge production [7,29,30]. This partially mitigated power imbalances by supporting active participation of diverse local stakeholders, including policymakers, community members, and businesses. These observations reflect broader analyses of governance asymmetries and power hierarchies in climate adaptation and health policy [30,31].

The fourth line of inquiry, transformative community engagement, is reflected in strategies that bridged research, policy, and civil society actors (Supplementary file 8). Consistent with previous work on transdisciplinary collaboration [9,32], our findings demonstrate that inclusive co-design and iterative feedback processes can generate counter-hegemonic knowledge, strengthen research–policy links, and support multi-sectoral action for complex health challenges [19]. These approaches not only enhance local ownership but also institutionalise participatory pathways for climate–health action in resource-constrained contexts.

Reflections on transdisciplinary processes for transformation (Supplementary file 9) revealed the inherent ‘tensions’ [33] in navigating power relations, fostering meaningful engagement, ensuring equitable knowledge co-production [34], and maintaining dialogue across disciplines [35]. The eight-phase TD approach strengthened trust, reflexivity, and collaboration across sites, exemplifying methodological innovation in contexts where institutional fragmentation and asymmetrical power relations often hinder systemic change [36]. This resonates with emerging literature on reflexive transdisciplinarity [37,38] which underscores iterative learning and boundary negotiation as mechanisms for transformative change in sustainability science.

In examining how transdisciplinary approaches can reveal and address the structural determinants underlying climate–NCD syndemics while challenging power dynamics through meaningful stakeholder engagement, we argue that TD engagement can transform governance relationships, amplify community agency, and advance equity-oriented climate–health responses. We propose a novel Integrated Syndemic Climate–Health Framework (ISCHF) that synthesises these insights into a practical tool, combining multifactorial hazard analysis, knowledge synthesis, stakeholder co-design, and iterative evaluation. It operationalises the five lines of inquiry by guiding multisectoral teams to map syndemic vulnerabilities, integrate health indicators into adaptation planning, and embed community participation in decision-making. In doing so, it bridges the gap between research and practice, demonstrating how transdisciplinary processes can generate actionable, equity-centred solutions for climate-resilient urban health systems in LMICs.

### A novel Integrated Syndemic Climate–Health Framework (ISCHF)

The framework is composed of six main components: Holistic Syndemic Analysis, Stakeholder-Driven Co-Design, Transdisciplinary Integration, Adaptive Implementation Strategies, Enhanced Communication and Capacity Building and Evaluation and Continuous Improvement. For each component, the framework provides two or three practical examples of interventions or actions that can be taken to achieve integrated solutions to solving climate and health challenges.

**Figure 3.**
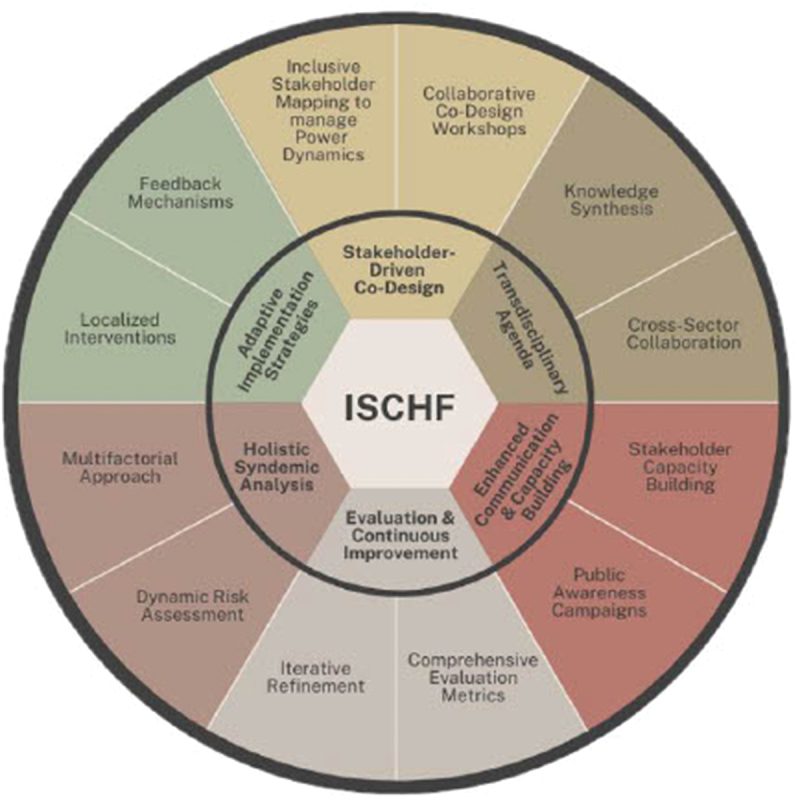
Novel Integrated Syndemic Climate–Health Framework (ISCHF).

Source: Author, 2024.

The ISCHF (Figure 3; Table 4) makes four distinctive contributions that address critical gaps in existing frameworks: First, it explicitly centers Global South urban contexts rather than adapting frameworks developed primarily for Global North settings, incorporating stakeholder perspectives on how climate–NCD interactions manifest differently across urban contexts with varying governance capacities, histories, and political economies. Second, the ISCHF responds to current critique [38] on providing practical and methodological guidance for transdisciplinary engagement that addresses power asymmetries in knowledge production, that elevate community perspectives and local knowledge systems. Third, by incorporating how private commercial sectors actors shape urban food and built environments, our framework explicitly addresses commercial determinants of health that other approaches often minimize and the inadequate attention set on corporate power in health frameworks [39]. Fourth, the ISCHF provides specific mechanisms for translating transdisciplinary knowledge into policy action through its ‘Adaptive Implementation Strategies’ component. This responds to the implementation gap identified in systematic reviews of planetary health frameworks [13,40] and the need to better integrate policy frameworks with environmental health strategies and practical interventions [9].

**Table 4.** Novel Integrated Syndemic Climate–Health Framework (ISCHF).

Holistic Syndemic Analysis	Multifactorial Approach	Incorporate a comprehensive analysis of multiple syndemic hazards, e.g. air pollution, food security, and urban density, to understand their combined impacts on health.
	Dynamic Risk Assessment	Employ advanced modelling techniques to simulate interactions between climate events and health outcomes, accounting for variability across urban contexts.
Stakeholder-Driven Co-Design	Inclusive Stakeholder Mapping and Collaborative Co-Design Workshops	Identify and engage a diverse range of stakeholders, including government agencies, civil society organisations, industry actors, academic institutions, and community representatives. Facilitate iterative workshops to co-create solutions, ensuring stakeholder input in every phase of the research and intervention process.
	Power Dynamics Management	Address and balance power dynamics to ensure equitable participation and decision-making.
Transdisciplinary Integration	Knowledge Synthesis	Integrate insights from various disciplines (e.g. public health, environmental science, urban planning) to form a unified understanding of climate–health interactions.
	Cross-Sector Collaboration	Foster collaborations across sectors to develop comprehensive strategies that address both health and environmental concerns.
Adaptive Implementation Strategies	Localised Interventions	Develop tailored interventions based on localised risk assessments and stakeholder inputs, addressing specific urban challenges and community needs.
	Feedback Mechanisms	Implement regular feedback loops to evaluate the effectiveness of interventions and adapt strategies as needed.
Enhanced Communication and Capacity Building	Stakeholder Capacity Building	Provide training and resources to stakeholders to enhance their understanding of syndemic health risks and effective intervention strategies.
	Public Awareness Campaigns	Raise awareness about the combined impacts of climate and health hazards through targeted communication strategies and community engagement.
Evaluation and Continuous Improvement	Comprehensive Evaluation Metrics	Develop and apply robust metrics to assess the impact of interventions on health outcomes and climate resilience.
	Iterative Refinement	Use evaluation findings to continuously refine the framework, ensuring that it remains responsive to emerging challenges and opportunities.

### Implications for policy and practice

We suggest several key areas for optimising policy and practice in the field of climate–NCD syndemics. Our research demonstrates the need for policy frameworks that explicitly recognize and address power asymmetries in climate–health governance. Urban planning policies should incorporate mechanisms for meaningful participation of marginalized communities, not merely as consultative stakeholders but as equal partners in decision-making processes. This requires institutional reforms that create formal spaces for counter-hegemonic knowledge to influence policy design, particularly in sectors controlling built and food environments. The ISCHF provides a practical mechanism to achieve this by guiding multisectoral teams to co-design localised interventions – such as mapping syndemic hotspots, embedding community feedback loops into urban planning, and integrating health indicators into climate-resilience policies, thereby operationalising the study’s findings to bridge the research–practice gap. We also describe the complex linkages between climate events and health impacts highlighted by stakeholders call for integrated policy approaches that transcend sectoral boundaries. Health ministries must collaborate with environmental, urban planning, and economic development departments to develop coordinated responses to syndemic hazards. Our multi-actor mapping provides a practical tool for identifying critical partnership opportunities across traditionally siloed domains. Finally, our findings on stakeholder engagement strategies suggest the need for transformative approaches to community participation in health governance. Policy frameworks should institutionalize processes for ongoing dialogue between community members, researchers, and policymakers, creating accountability mechanisms that ensure community knowledge informs implementation. For practitioners, our research offers practical guidance for operationalizing transdisciplinary approaches in resource-constrained settings. The documented lessons learned across eight phases of TD research can inform more equitable stakeholder engagement practices. We recommend that practitioners critically examine power dynamics within their engagement processes, explicitly addressing hierarchical concerns through intentional design of collaborative and inclusive spaces.

These implications align with calls for health systems strengthening in LMICs to address upstream determinants of NCDs while building climate resilience [41–43]. By centering equity concerns and power analysis in climate–health responses, policies and practices can more effectively address the structural drivers of health inequities in rapidly urbanizing contexts facing syndemic threats.

### Strengths and limitations

This study’s main strengths include its novel integration of critical theory with transdisciplinary practice across diverse LMIC urban contexts, offering practical methodological guidance while centering structural determinants often neglected in climate–health frameworks. The engagement of diverse stakeholders across sectors created unprecedented spaces for interaction and knowledge co-production. Key limitations include challenges in achieving balanced stakeholder representation, with some sectors proving difficult to engage consistently. The inclusion of some older references also reflects the scarcity of recent empirical literature on climate–NCD syndemics and transdisciplinary implementation in LMIC contexts. Additionally, the translation from conceptual framework to sustainable action remains challenging given identified power disparities. Finally, while the multi-site approach enhances comparative insights, contextual specificities may limit transferability to other urban settings.

## Conclusion

Our research demonstrates how transdisciplinary approaches can uncover the structural, social, and political determinants underpinning the climate–NCD syndemic while explicitly addressing power dynamics through meaningful stakeholder engagement. By creating spaces for counter-hegemonic knowledge production, transforming governance relationships, and illuminating climate–health equity dimensions, our approach advances both theoretical and applied understanding of syndemic interactions. The Integrated Syndemic Climate–Health Framework (ISCHF) offers a transformative, equity-centred pathway for tackling climate–NCD challenges in urban LMIC contexts through multifactorial hazard analysis, co-designed knowledge synthesis, and iterative evaluation. Future research should sustain transdisciplinary collaborations, assess long-term impacts, and refine engagement practices that challenge entrenched power structures while advancing planetary health equity.

## Supplementary Material

All_Supplementary_files_word_doc_docx_clean.docx

Supplementary file 3 _DataExtraction_excel.xlsx

Checklist_Good_Reporting_of_A_Mixed_Methods_Study_GAMMS_d.docx

## Data Availability

The qualitative data generated through stakeholder workshops contain sensitive information and cannot be made publicly available due to ethical considerations and confidentiality agreements with participants. Anonymized summaries of workshop findings are available from the corresponding author upon reasonable request. The methodological tools developed for stakeholder engagement, including workshop guides and analysis frameworks, are available as supplementary materials to this article.

## References

[1] Yadav UN, Rayamajhee B, Mistry SK, et al. A syndemic perspective on the management of non-communicable diseases amid the COVID-19 pandemic in low-and middle-income countries. Front Public Health. 2020;8:508. doi: 10.3389/fpubh.2020.00508 33102414 PMC7545493

[2] Mendenhall E, Kohrt BA, Norris SA, et al. Non-communicable disease syndemics: poverty, depression, and diabetes among low-income populations. Lancet. 2017;389:951–15. doi: 10.1016/S0140-6736(17)30402-6 28271846 PMC5491333

[3] Tsai AC, Mendenhall E, Trostle JA, et al. Co-occurring epidemics, syndemics, and population health. Lancet. 2017;389:978–982. doi: 10.1016/S0140-6736(17)30403-8 28271848 PMC5972361

[4] Kundu D, Pandey AK. World urbanisation: trends and patterns. In: Kundu D, Sietchiping R, Kinyanjui M, editors. Developing national urban policies: ways forward to green and smart cities. Springer; 2020. p. 13–49.

[5] Projections of mortality and causes of death. 2015 and 2030. [Internet]. Available from: http://www.who.int/healthinfo/global_burden_disease/projections/en/

[6] Gouda HN, Charlson F, Sorsdahl K, et al. Burden of non-communicable diseases in Sub-Saharan Africa, 1990–2017: results from the Global Burden of Disease Study 2017. Lancet Glob Health. 2019;7(10):e1375–e87. doi: 10.1016/S2214-109X(19)30374-2 31537368

[7] Lang DJ, Wiek A, Bergmann M, et al. Transdisciplinary research in sustainability science: practice, principles, and challenges. Sustain Sci. 2012;7:25–43. doi: 10.1007/s11625-011-0149-x

[8] Knapp CN, Reid RS, Fernández-Giménez ME, et al. Placing transdisciplinarity in context: a review of approaches to connect scholars, society and action. Sustainability. 2019;11:4899. doi: 10.3390/su11184899

[9] Wardani J, Bos JJ, Ramirez-Lovering D, et al. Enabling transdisciplinary research collaboration for planetary health: insights from practice at the environment-health-development nexus. Sustain Devel. 2022;30:375–392. doi: 10.1002/sd.2280

[10] Osofsky SA, Pongsiri MJ. Operationalising planetary health as a game-changing paradigm: health impact assessments are key. Lancet Planet Health. 2018;2:e54–e5. doi: 10.1016/S2542-5196(17)30183-3 29615233

[11] Oni T, Assah F, Erzse A, et al. The global diet and activity research (GDAR) network: a global public health partnership to address upstream NCD risk factors in urban low and middle-income contexts. Global Health. 2020;16:1–11. doi: 10.1186/s12992-020-00630-y 33076935 PMC7570103

[12] Whitmee S, Haines A, Beyrer C, et al. Safeguarding human health in the Anthropocene epoch: report of the Rockefeller Foundation–Lancet Commission on planetary health. Lancet. 2015;386:1973–2028. doi: 10.1016/S0140-6736(15)60901-1 26188744

[13] Pongsiri MJ, Bassi AM. A systems understanding underpins actions at the climate and health nexus. Int J Environ Res Public Health. 2021;18:2398. doi: 10.3390/ijerph18052398 33804531 PMC7967726

[14] Zinsstag J, Schelling E, Crump L, et al. One health: the theory and practice of integrated health approaches. Cabi; 2020.

[15] Kingsley P, Taylor E. One health: competing perspectives in an emerging field. Parasitology. 2017;144:7–14. doi: 10.1017/S0031182015001845 26817944

[16] Krieger N. Epidemiology and the people’s health: theory and context. Oxford University Press; 2024.

[17] Singer M, Bulled N, Ostrach B, et al. Syndemics and the biosocial conception of health. Lancet. 2017;389:941–950. doi: 10.1016/S0140-6736(17)30003-X 28271845

[18] Pineo H, Zimmermann N, Cosgrave E, et al. Promoting a healthy cities agenda through indicators: development of a global urban environment and health index. Cities Health. 2018;2:27–45. doi: 10.1080/23748834.2018.1429180

[19] Pineo H, Turnbull ER, Davies M, et al. A new transdisciplinary research model to investigate and improve the health of the public. Health Promot Int. 2021;36:481–492. doi: 10.1093/heapro/daaa125 33450013 PMC8049543

[20] Wardani J, Bos JJA, Ramirez-Lovering D, et al. Towards a practice framework for transdisciplinary collaboration in planetary health. Glob Sustain. 2024;7:e16. doi: 10.1017/sus.2024.6

[21] Bromham L, Dinnage R, Hua X. Interdisciplinary research has consistently lower funding success. Nature. 2016;534:684–687. doi: 10.1038/nature18315 27357795

[22] WHO UH. Global report on urban health: equitable, healthier cities for sustainable development. Geneva, Switzerland: World Health Organization; 2016.

[23] Ebi KL, Harris F, Sioen GB, et al. Transdisciplinary research priorities for human and planetary health in the context of the 2030 agenda for sustainable development. Int J Environ Res Public Health. 2020;17:8890. doi: 10.3390/ijerph17238890 33265908 PMC7729495

[24] Loewenson R, Godt S, Kapata PC. Framings of and priorities for action on the commercial determinants of health in Sub-Saharan Africa. Training and Research Support Centre. 2021.

[25] Zhao Q, Guo Y, Ye T, et al. Global, regional, and national burden of mortality associated with non-optimal ambient temperatures from 2000 to 2019: a three-stage modelling study. Lancet Planet Health. 2021;5:e415–e25. 34245712 10.1016/S2542-5196(21)00081-4

[26] Frumkin H, Haines A. Global environmental change and noncommunicable disease risks. Annu Rev Public Health. 2019;40:261–282. doi: 10.1146/annurev-publhealth-040218-043706 30633714

[27] Galea S, Link BG. Six paths for the future of social epidemiology. Am J Epidemiol. 2013;178:843–849. doi: 10.1093/aje/kwt148 24008899 PMC3775546

[28] Eriksen S, Schipper ELF, Scoville-Simonds M, et al. Adaptation interventions and their effect on vulnerability in developing countries: help, hindrance or irrelevance? World Dev. 2021;141:105383. doi: 10.1016/j.worlddev.2020.105383

[29] Brisbois B, Shmelev S. Ecosystem approaches to health and knowledge-to-action: towards a political ecology of applied health-environment knowledge. J Polit Ecol. 2017;24. doi: 10.2458/v24i1.20961

[30] Nightingale AJ. Power and politics in climate change adaptation efforts: struggles over authority and recognition in the context of political instability. Geoforum. 2017;84:11–20. doi: 10.1016/j.geoforum.2017.05.011

[31] Chu E, Anguelovski I, Roberts D. Climate adaptation as strategic urbanism: assessing opportunities and uncertainties for equity and inclusive development in cities. Cities. 2017;60:378–387. doi: 10.1016/j.cities.2016.10.016

[32] Angelstam P, Khaulyak O, Yamelynets T, et al. Green infrastructure development at European Union’s eastern border: effects of road infrastructure and forest habitat loss. J Environ Manage. 2017;193:300–311. 28232244 10.1016/j.jenvman.2017.02.017

[33] Clark MB, Wrzesinski T, Garcia AB, et al. Long-read sequencing reveals the complex splicing profile of the psychiatric risk gene CACNA1C in human brain. Mol Psychiatry. 2020;25:37–47. doi: 10.1038/s41380-019-0583-1 31695164 PMC6906184

[34] Angelstam P, Andersson K, Annerstedt M, et al. Solving problems in social–ecological systems: definition, practice and barriers of transdisciplinary research. Ambio. 2013;42:254–265. doi: 10.1007/s13280-012-0372-4 23475660 PMC3593036

[35] Malmqvist E, Oudin A. Bridging disciplines-key to success when implementing planetary health in medical training curricula. Front Public Health. 2024;12:1454729. doi: 10.3389/fpubh.2024.1454729 39165783 PMC11333318

[36] Siltanen U, Prass M, Puhakka R, et al. Promoting planetary health at regional level: a qualitative analysis of a multisectoral health and environment program. One Health Cases. 2024;2024:ohcs20240005. doi: 10.1079/onehealthcases.2024.0005

[37] Fazey I, Schäpke N, Caniglia G, et al. Transforming knowledge systems for life on earth: visions of future systems and how to get there. Energy Res Soc Sci. 2020;70:101724. doi: 10.1016/j.erss.2020.101724

[38] Popa F, Guillermin M, Dedeurwaerdere T. A pragmatist approach to transdisciplinarity in sustainability research: from complex systems theory to reflexive science. Futures. 2015;65:45–56. doi: 10.1016/j.futures.2014.02.002

[39] McKee M, Stuckler D. Revisiting the corporate and commercial determinants of health. Am J Public Health. 2018;108:1167–1170. doi: 10.2105/AJPH.2018.304510 30024808 PMC6085040

[40] Pongsiri MJ, Gatzweiler FW, Bassi AM, et al. The need for a systems approach to planetary health. Lancet Planet Health. 2017;1:e257–e9. 29851620 10.1016/S2542-5196(17)30116-X

[41] Haines A, Ebi K. The imperative for climate action to protect health. N Engl J Med. 2019;380:263–273. doi: 10.1056/NEJMra1807873 30650330

[42] Chersich MF, Wright CY, Venter F, et al. Impacts of climate change on health and wellbeing in South Africa. Int J Environ Res Public Health. 2018;15:1884. doi: 10.3390/ijerph15091884 30200277 PMC6164733

[43] Rasanathan K, Bennett S, Atkins V, et al. Governing multisectoral action for health in low-and middle-income countries. PLoS Med. 2017;14:e1002285. doi: 10.1371/journal.pmed.1002285 28441387 PMC5404752

