# Supplementary material for: Urban climate–NCD syndemics in LMICs: a transdisciplinary framework for Health action
Source: Glob Health Action. 2026 Apr 22;19(1):2650971. doi: 10.1080/16549716.2026.2650971 (PMC13104006; doi:10.1080/16549716.2026.2650971)
Supplement: All_Supplementary_files_word_doc_docx_clean.docx [file ZGHA_A_2650971_SM7268.docx]

**Supplementary File 1: Detailed description of Stakeholder Workshops**

The workshops were structured using harmonized methodology and agenda (see supplementary material) across sites. The agenda of the workshops consisted of a welcome session and a general presentation of GDAR, followed by three technical work sessions on (1) syndemic variables, (2) health impacts of climate change events, and (3) policy and actor mapping. While the harmonized agenda provided a roadmap for the critical components of the workshop, it also provided flexibility for each team to adapt and cater to different research and stakeholder engagement needs during planning or implementation. The agenda consisted of:

- presentations to ensure that all stakeholders understand the main objectives, concepts, and workshop methodology and to cover any needed explanation and to cover any needed explanation for non-scientific stakeholders.
- interactive exercises using interactive platforms (e.g. mentimeter: [www.mentimeter.org](http://www.mentimeter.org) , cards, and whiteboards for collective exercises and setting working groups for more targeted suggestions.
- plenary sessions for group feedback and comparative findings.
- case studies to showcase 1 or 2 inspiring case studies illustrating outcomes from participatory research or having used multi-stakeholder approaches.

if needed, pre- and post-workshop surveys were sent in advance or after the workshop to provide definitions, assess stakeholder needs, and consider how different issues are understood and tackled in the local context.

The different phases of the stakeholder engagement workshops are presented in the figure below.


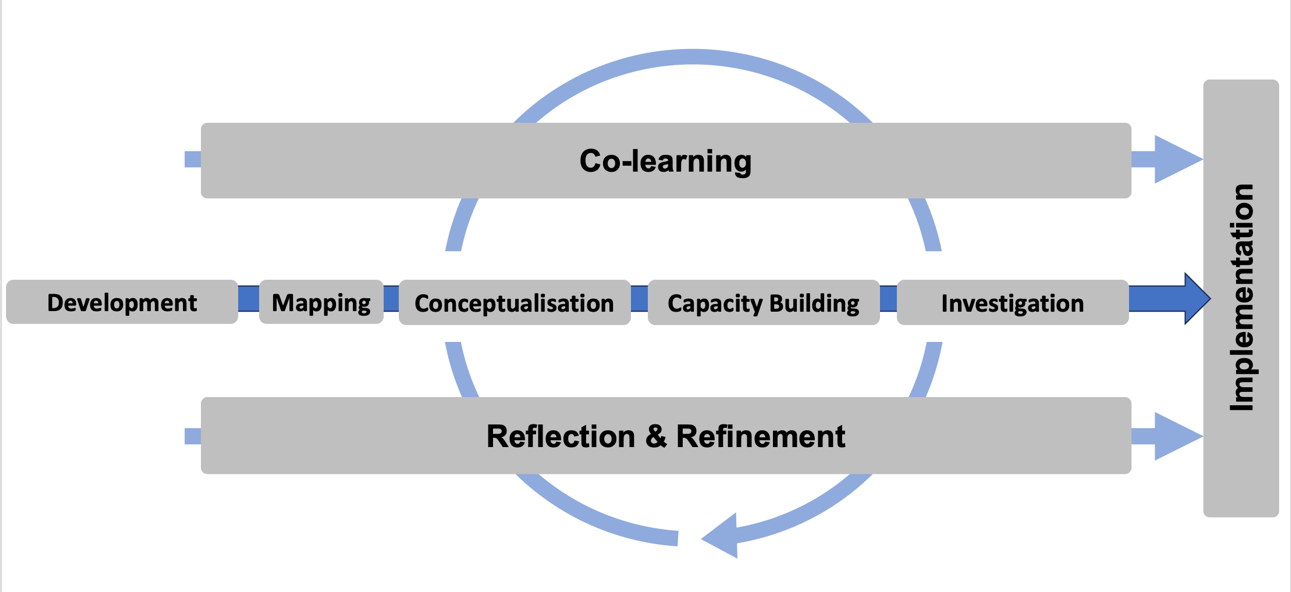


**Development and conceptualization phases**

To pre-develop and conceptualize the stakeholder engagement workshops, the research team held several co-creation meetings and four working sessions with members from all the country sites, and incorporated inputs. With expertise from senior principal investigators and local team experiences, we brainstormed the best methods to address syndemic hazards in different sites. The research teams had diverse expertise on the syndemic hazards and TD application. During the working sessions, we set up the process to structure the meetings for planning, strategy, feedback, and experience-sharing across sites. We generated harmonized ethical protocols to ensure rigorous data collection during the workshops and designed a training module for all researchers and workshop facilitators. We used various tools from the technical Biodiversa Handbook site ([www.Biodiversa.org](http://www.biodiversa.org)) and the td-net toolbox (https://naturalsciences.ch/co-producing-knowledge-explained/methods/td-net_toolbox) to inform our discussions. At the end of the four working sessions, we 1) developed a list of specific objectives (aligned with values, principles and outcomes that would drive our approach) to inform the workshops and 2) laid out a framework for the design process. The pre-development and conceptualization phases were useful in gaining consensus on the workshops' specific objectives and activities (Table 1), as well as the agendas.

**Capacity building phase**

To equip the GDAR team for the workshops, a 3-phased training module was organised for the researchers. This was necessary because, despite extensive experience with engaging stakeholders across the network, the introduction of a new engagement framework revealed significant variations in capacity. Therefore, training was essential to ensure a consistent approach and to align all parties with the updated standards and expectations. The training combined lectures and hands-on experiential exercises on the theoretical understanding of syndemic variables and practical methods to engage stakeholders on the topic. For example, we prepared syndemic variables memory cards and traffic-light systems to structure the activities and prepared the researchers to run the workshop. In the training session, researchers were provided scenarios to explore the impacts of syndemic hazards on health and convey their insights on diet and physical activity. They also discussed the implications of different climate events from a set list of context-specific and real-time events selected from different sites. Finally, the researchers were trained in actor constellation methods, where they had to identify key actors, their backgrounds, and the power relationships between them.

These training activities were used to generate perspectives on how best to elicit identifying drivers of the syndemic hazards conditions underpinning the effects of syndemic hazards on diet and physical activity and to motivate a discussion around how to adapt the exercises for use in the workshops with stakeholders who had experience and knowledge in the field of climate, health, physical activity, and diet in LMICs. The training also led to the co-development of a facilitation manual for the workshop and communication materials for the larger team and dissemination. After the training, each site identified team members to serve key roles during the workshop. These roles included the hosting from the PI from each site, a site coordinator, three researchers who assisted with the training, and at least two rapporteurs and two note takers. Each team was responsible for identifying (via stakeholders mapping) and contacting participants at least one month before the workshop using their preferred communication methods. The facilitation of the training was led by members of the research team with expertise in environmental exposures to health, spatial analysis of syndemic variables and methodological expertise in TD participatory methods.

**Mapping phase**

The stakeholder mapping and analysis were conducted using the validated stakeholder analysis methodology from the Biodiversa toolkit methodology [13] in collaboration with workshop facilitators and team leads from each study site ([www.Biodiversa.org](http://www.biodiversa.org)). During the stakeholder mapping and analysis phase, research teams reflected on the aims and objectives of the project. They identified the relevant stakeholders who can 1) contribute or change the situation under investigation, 2) generate new flows of knowledge and/or 3) focus on policy processes. As outlined in the Biodiversa toolkit, stakeholder mapping and analysis were conducted in four main stages:

- Develop a stakeholder matrix by core sector and stakeholder type
- Categorize stakeholders by power and influence
- Define the current collaboration status with each stakeholder
- Identify a core stakeholder advisory group for GDAR

The mapping process by each country team generated a list of relevant, diverse stakeholders (master list) that were categorized by interest and power influence. The list categorized stakeholders into six different actor typologies: 1) Policy, 2) commercial, 3) community, 4) not-for-profit, 5) academia and technicians and 6) others. The list of sectors covered by all the stakeholders is provided in box 2.

*Box 1: List of identified sectors relevant to the syndemic hazards in the GDAR country sites*

| Agriculture  City work/ administration  Climate Change  Agriculture  City work/ administration  Climate Change  Education  Environment  Finance & Economic Development | Food & Nutrition  Housing & Informal settlements  International development  Mining  Governance and Policy  Public health  Religion  Research | Retail  Social justice  Sports and Physical activity  Telecommunications/Media  Transportation and Urban Infrastructure  Urban Planning & Land Use  Youth development |
| --- | --- | --- |

After that, a senior researcher analysed the master list to assess stakeholder representativity across sectors, groups, and sites. A mini-stakeholder analysis workshop was then organized in February 2022 to share the results with the GDAR team and recommendations for Stakeholder engagement workshops participant identification (see following section) to optimize participant recruitment in sectors under-represented in the master list. Once the teams validated the master list, participants were identified based on their expertise in relevant sectors (box 1) as well as their belonging to three broad groups (policy, commercial and community actors). The stakeholders included volunteer adults across different socio-economic and demographic groups, pertaining to different interest groups, with different expertise, power, and influence concerning the project. For each workshop, an effort was made to include at least 10 participants from climate, PA, and diet and participants from diverse institutions (e.g., academia and not-for-profit). Each workshop was structured to engage approximately thirty stakeholders. Each participant received an invitation via telephone, electronic messaging or in person where required. They were also provided with information on the GDAR study and the purpose of the workshops, along with a short introductory video in the national language of the study site (https://www.youtube.com/watch?v=i9iCjDGQ6Yw).

**Investigation phase**

All workshops were full-day sessions and were conducted in person. Each study site held its workshop in their native language (i.e., English, Swahili, French, or Portuguese), using communication that fits the audience and matched the study purpose. The teams also adjusted to locally relevant climate hazards and the audience’s specificities. The teams used a facilitators’ guide to facilitate the session (See Supplementary material 2) We employed an iterative approach where the structure of each workshop was slightly altered based on context, experiences and lessons reported from preceding workshops. All sites generated media material (e.g. video) for promotion and awareness during and after the Stakeholder engagement workshops. All sites reported live updates on social media (e.g. Twitter). After each workshop, all sites shared their experiences and provided feedback on the process and materials used. All workshop attendance and activities were reported in standardized registration lists, with a full report and a series of standardized templates. The reports were prepared by each study site and included background about the study, the objectives guiding the workshops, a summary agenda, the findings from the discussion with participants during the workshop, action ideas, and reflections and insights that emerged from the workshop.

**Implementation phase**

The preparation of this manuscript marks the initial step in the implementation phase of GDAR's larger program of work, leveraging new knowledge generated from the workshops to address societal challenges. The knowledge generated from the stakeholder engagement workshops is currently being utilized to inform and guide the subsequent phases of the program of work including policy analyses phases and design of interventions. This foundational understanding is being integrated into the design and implementation of ongoing activities, ensuring that the insights and lessons learned are applied to optimize strategies, enhance stakeholder engagement, and address key challenges across the program.

The workshops consisted of five main activities aligned with the five key lines of inquiry of the paper and explained in more details here:

**Activity 1:** Identify and discuss relevant syndemic hazards.

The activity is designed to inform environmental audits of syndemic variables contributing to unhealthy diets, physical inactivity, and health risks. In this activity, the stakeholders discussed the syndemic variables they considered relevant and available for assessing their respective environments. The variables were classified into three broad categories: climate, physical environment, and food.

**Activity 2:** List key climate events from the last 10 years and discuss their impact on health.

In this activity, climate change events are used as a lens through which policies and interventions related to health can be examined and how policy and community initiatives are triggered as a response to specific climate events. The participants were invited to identify relevant climate change events in the last 10 years in different sites to discuss their impacts on health. The groups discussed and listed relevant climate change events (see Table x) and their effects on health, ranking them by order of importance. Each site focused on relevant local and most recurrent climate events, which they listed and ranked by order of importance (see Table x). The rapporteur then took notes from the discussion on how participants perceived these events to affect their health. We applied thematic analysis methods to analyze the note transcripts. The analysis resulted in 7 thematic groups representing the health impacts of different climate events as perceived by the participants.

**Activity 3:** Identify key local actors across critical sectors to respond to climate events.

This activity focuses on the GDAR component to better understand how community, policy, and commercial actors (and their respective institutions/groups) respond, cope, and adapt to climate change shocks. The groups chose one of the climate change events they listed in the previous activity and described what coping strategies were ongoing or planned to address the effects of the event. They proposed a list of actors from community, policy and commercial backgrounds, involved at different levels and with specific roles to cope with the listed climate change events and related public policies.

**Activity 4:** Instigate discussion and feedback on the existing approach to the Theory of Change.

This activity focuses on the GDAR theory of change (ToC) per the academic research proposal. The participants discussed the expected changes during the research and what inputs, activities, outputs, outcomes, and impacts drive the study. The discussion also covered the critical underlying assumptions that must be met to bring about change at the societal level.

**Activity 5:** Identify community engagement and involvement (CEI) strategies.

This activity acknowledges Community Engagement and Involvement (CEI) as a core component of GDAR research and the importance of undertaking the research in collaboration with the groups most likely affected by the research outcomes. Such groups include but are not limited to national officers, research partners, policymakers, local communities’ representatives, and public managers. In anticipation of future GDAR participation, participants brainstormed on ways to maintain community engagement initiatives they would like to support.

**Supplementary File 2: Facilitator Guide for Stakeholder Workshops**

FACILITATOR GUIDE

GDAR

STAKEHOLDER CONSULTATION NO.1

YEAR 2022

Note: This document is intended for the GDAR facilitator to use when delivering the first stakeholder consultation session to their assigned sites. It contains a run down for each section, including objectives, expected output, materials and recommended exercises. It is not to be distributed to participants.

**TABLE OF CONTENTS**

[SECTION I: GENERAL NOTES 3](#_heading=h.30j0zll)

[SECTION II: AGENDA 4](#_heading=h.1fob9te)

[SECTION III: FACILITATION GUIDELINES 5](#_heading=h.3znysh7)

[Session 0 “Welcome” 5](#_heading=h.2et92p0)

[Session 1 “Introduction” 6](#_heading=h.tyjcwt)

[Session 2 Study Variables 7](#_heading=h.3dy6vkm)

[Session 2A Rate study variables 8](#_heading=h.1t3h5sf)

[Session 2B Find climate change events 9](#_heading=h.4d34og8)

[Session 3 Find key actors 10](#_heading=h.2s8eyo1)

[Session 4 Community engagement strategies 11](#_heading=h.17dp8vu)

[Session 5 Study design 11](#_heading=h.3rdcrjn)

[Session 6 Concluding remarks 12](#_heading=h.26in1rg)

[SECTION IV: GENERAL FACILITATION SKILLS 14](#_heading=h.lnxbz9)

# SECTION I: GENERAL NOTES

A: Overarching Goal
This consultation is designed to identify and understand the priorities of diverse stakeholders involved in GDAR spaces, and to incorporate their perspectives in study objectives and design. The session will also set foundations for engagement throughout the GDAR Spaces project.

B: Key Understandings
Participants provide input on different variables that impact health through changes in the environment and climate. Participants will also provide insight on different actors which they believe are important in responding to climate change events and in building environmental and climate policies to safeguard health. This guide is designed to provide support to facilitators of this session.

C: Objectives of stakeholder consultation no.1

- Identify and validate variables that link environment and climate change to health
- Identify sources where data on these variables can be collected
- Identify and rank key climate change events (last 10 years)
- Identify key local actors across sectors that are critical to respond to climate events
- Identify community engagement strategies used to respond to climate events
- Provide feedback on existing Theory of Change (ToC) of GDAR spaces

D: Session Materials

- Facilitator Guide
- Handouts
- Computer with Internet access
- Projector
- Sign-in sheet
- PowerPoint
- Chart paper
- Markers
- Flip charts
- Sticky notes

# SECTION II: AGENDA

| Time | Session | Activity | Key points | Equipment/Materials |
| --- | --- | --- | --- | --- |
| - | Registration |  | Break out groups sorted | Flip chart/ Registration list/  Consent form |
| 5 min | 0- Welcome | Plenary | Greetings  Housekeeping rules | Presentation template |
| 30 min | 1- Introduction | Plenary | GDAR network and GDAR Spaces  The rundown of the workshop  Icebreaker | Presentation template  List of breakout groups |
| 30 min | 2- Study Variables | Plenary | Introduce different variables impacting on health in the context of environmental and climate change  Present 1 case study | Presentation template |
| 40 min | 2A- Rate the Variables | Small groups  Plenary | Rate the variables  Identify the sources of data  Report if any variables are missing | Cards  List of data sources  Reporting template |
| TEA & CAKE BREAK | | | | |
| 40 min | 2B- Find climate change events | Small groups  Plenary | Present a short list of climate change events  Select the events that have happened in last 10 years  Rank events by importance  Explain why they are important | A ‘menu’ of climate change events  Reporting template |
| 40 min | 3- Find Key actors | Plenary | Identify sectors of relevance  Identify new actors by sector  Mapping the connection between actors | Example of actors  Example of Map for connecting actors  Reporting template |
| LUNCH BREAK | | | | |
| 45 min | 4- Community engagement strategies | Plenary | Identify existing CEI strategies  Rank CEI by importance  Explain why they are important | List of CEI strategies  Reporting template |
| 60 min | 5- Theory of Change | Small groups | Present Theory of Change (ToC) used in initial proposal  Gather input of ToC through interactive discussion | Presentation template of ToC |
| 15 min | 6- Closing remarks | Plenary | Thank you note  Summary of the workshop  Action points and way forward |  |

(Note: The agenda is intended for a full day session; however, you may need to accommodate accordingly.)

# SECTION III: FACILITATION GUIDELINES

| Session 0 “Welcome” |
| --- |
| Objectives:   - To register attendees and have their consents page signed; - To have an overview of the sectors and main interested of the people that attended; - To create and inform people about the breakout groups (where applicable); - To allow participants a quick reflexion and light introduction to GDAR network with video |
| Considerations for different sites:   - Breakfast during registration - Boards to place attendees - Organisation of break out groups and colour coding upfront (e.g. by area of work, level of expertise) - Flexibility on starting time |

| Expected outputs | Checked (yes/no) |
| --- | --- |
| Electronic list of attendees |  |
| Sign all consents sheets |  |
| Map of sectors and areas of interest |  |
| List of breakout groups |  |
| Survey results |  |

| Supporting Materials | Checked (yes/no) |
| --- | --- |
| Excel template on computer or paper based |  |
| Hand, paper and after scan, electronic signatures. |  |
| Flip chart with post-its |  |
| Excel template or paper based |  |

| Instructions for mapping participants’ sectors and areas of interest |
| --- |
| White board, post-it and markers  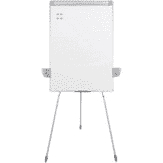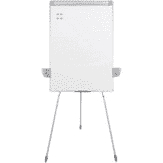 |
| Board 1 Instruction: Write your name in the post-it and place it in the circle which most defines your area of work.  Circle names: Food, Physical Activity, Climate change  Board 2 Instruction: Write down your name and what is the type of work you do  Circle names: Policy, Data collection, community engagement |

| Tips about this session |
| --- |
| Housekeeping rules need to be expressed with sensitivity - considering different levels of authority of stakeholders |

| Session 1 “Introduction” |
| --- |
| Objectives:   - To introduce GDAR network, projects and GDAR Spaces. - To present the rundown of the workshop (overview of the activities and description of interactive sessions of the day) - To run an interactive exercise to introduce participants and align expectations - To iterate intention to build a core working group and engage further |
| Considerations for different sites:   - This session is to introduce and explain the project, outline the study approach and priorities and slides can be adapt to your site |

| Expected outputs | There are no expected outputs for this session, however, this session will be very helpful for you to set the stage for the overall workshop. |
| --- | --- |

| Supporting Materials | Checked (yes/no) |
| --- | --- |
| Greeting and housekeeping powerpoint template |  |
| GDAR spaces powerpoint template |  |
| GDAR teaser video |  |

| Instructions for the Ice breaker exercise |
| --- |
| Option 1) Free discussion by talking to the person next to each other   - Share with the person next to you: - Your name - Your area of work - Your main areas of interest (e.g. build environment, food, physical activity) - How do you envision yourself to be part of GDAR Spaces   Option 2) Randomly select 4 people to share their 3 points and the 3 points from the 2 persons next to them |
| Option 2) Mentimeter   - With the ‘open questions’ feature and ask: - Your name - Your area of work - Your main area of interest (e.g. build environment, food, physical activity) |

| Tips about this session |
| --- |
| Here, we explain what the workshop is about.  It is important that participants understand how their contributions will lead to tangible outputs & delivery of the project. They need to be active!  The ice breaker is an opportunity to scope where people are coming from, identifying roles, and showing inclusivity. We use this session to state and ensure that everyone’s opinion is considered equal. State that we will be looking for a core group to take work further and invite people to sign up. |

| Session 2 Study Variables |
| --- |
| Objectives:   - Introduce the background and the different variables impacting on health in the context of environmental and climate change |
| Considerations for different sites:   - A case study from your country or a similar site can be presented for participants to identify with |

| Expected outputs | Checked (yes/no) |
| --- | --- |
| Providing an introduction for participants to be able to enga Session 2A |  |

| Supporting Materials | Checked (yes/no) |
| --- | --- |
| Variables and climate change powerpoint template |  |

| Tips about this session |
| --- |
| This session needs to be in plain language and concise focusing on the interaction with and between participants.  Not all stakeholders will have interest to participate here for 2A and 2B, so it is to ensure breakout groups are split strategically. |

| Session 2A Rate study variables |
| --- |
| Objectives:   - To identify and rate the variables impacting on health in the context of environmental and climate change, and listing the different sources where data on these variables can be found |
| Considerations for different sites:   - Do break out groups that are small and moderated - After break out groups, plenary sessions should be long enough but this is flexible across sites (15mins) |

| Expected outputs | Checked (yes/no) |
| --- | --- |
| List of validated variables |  |
| List of data sources for each validated variable |  |
| List of contacts to seek and obtain the data |  |
| List of missing variables (if any) |  |

| Supporting Materials | Checked (yes/no) |
| --- | --- |
| Template presentation with short instruction on exercise |  |
| Cards |  |
| List of data sources |  |
| Reporting template |  |

| Instructions for exercise |
| --- |
| Option 1) Open discussion or  Option 2) Mentimeter |
| Relevant questions for facilitator to ask:   - Can you run through each variable on the cards? - Can you rate (validate/discard) each variable using a yes/no system or a traffic light system? - For each validated variable, can you link it to a data source? - Can you name the data source and if possible the person to contact in order to access the data ? - Are there any variables missing from the cards? If yes, which ones? |
| 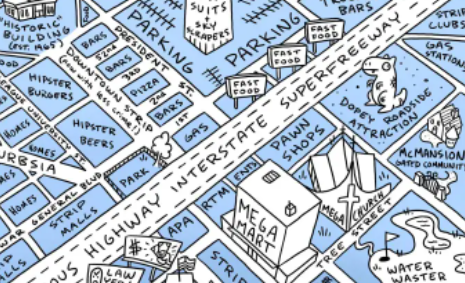  Printed map in a A2 page (one peer group)  Use a city map for interactive proposes and illustration of links between city and  The map can be from your city or from a cartoon. |
| SYNDETIC CONSTRUCTS CARDS  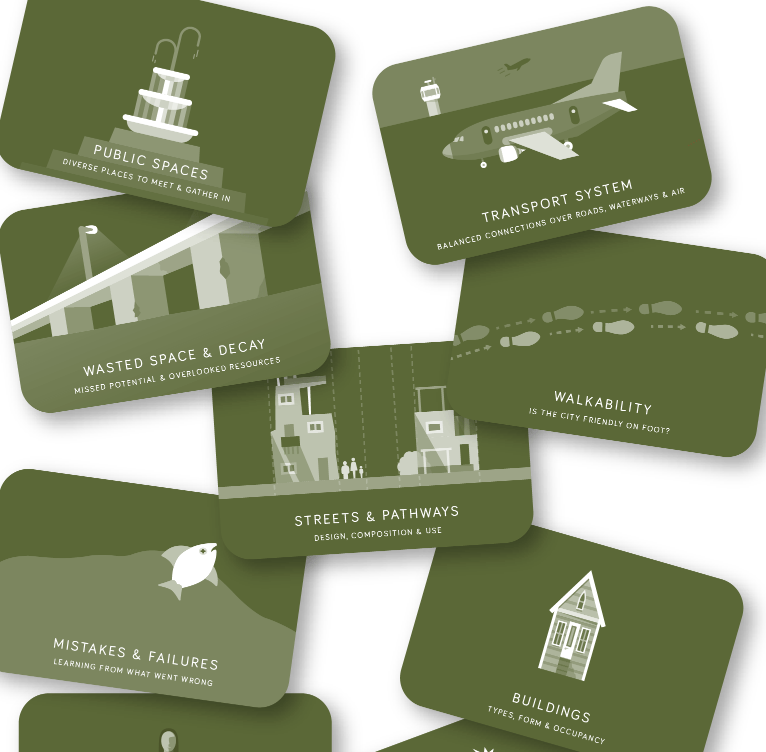  We suggest we use a card for each construct in order to make it more visibly helpful.  We are at the moment preparing the cards and would make them available for you to use  You would need to print the cards (depending on how you would use them, you can print 1 set or more sets of cards) |
| 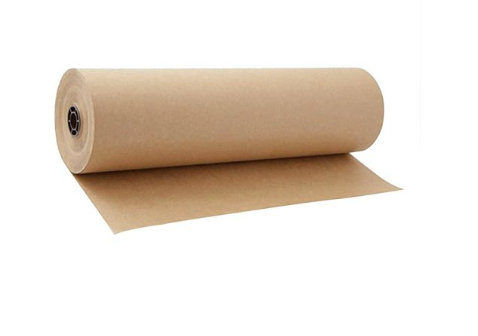  Paper for being placed in tables. |

| Tips about this session |
| --- |
|  |

| Session 2B Find climate change events |
| --- |
| Objectives:   - To identify and list climate change events that have occurred in the last 10 years and rank by order of importance |
| Considerations for different sites:   - It is preferable that facilitator is aware of WP7 objectives and able to explain to participants why they are invited to select and identify climate related events - Do break out groups that are small and moderated - After break out groups, plenary sessions should be long enough but this is flexible across sites (15mins) |

| Expected outputs | Checked (yes/no) |
| --- | --- |
| List of climate change events occurred in the last 10 years |  |
| Explanation of why these climate change events are important |  |

| Supporting Materials | Checked (yes/no) |
| --- | --- |
| A ‘menu’ of climate change events |  |
| Reporting template |  |

| Instructions for exercise |
| --- |
| Relevant questions for facilitator to ask:   - Can you recognise the climate change event on the ‘menu’? - Can you select the events that have occurred in the last 10 years? - Can you rate (validate/discard) each event using a yes/no system or a traffic light system? - Can you confirm that this event has had good public coverage in order to collect enough data for the research? - Can you rank the validated events by order of importance? - Can you describe the impact the event on diet and physical activity within communities? |

| Tips about this session |
| --- |
|  |

| Session 3 Find key actors |
| --- |
| Objectives:   - To identify, list and map key local policy, commercial and community actors by sectors of interest |
| Considerations for different sites:   - It is helpful if facilitator is aware of related objectives WP5, 6, 7 - Important to explain why we are interested in identifying policy, commercial, community actors and understand the links between these actors - Keep the same breakout groups as before - Flexibility here - each site can do break out groups if they decide |

| Expected outputs | Checked (yes/no) |
| --- | --- |
| List (names and contacts) of actors by sector of interest |  |
| Map of different actors and how they are connected to each other |  |

| Supporting Materials | Checked (yes/no) |
| --- | --- |
| Examples of different actors and sectors |  |
| Blank page for mapping and connecting actors |  |
| Reporting template |  |

| Instructions for exercise |
| --- |
| Relevant questions for facilitator to ask:   - Can you suggest the key sectors you think are critical in responding to climate events? - Who are the key actors (individuals, networks, organisations, state departments) critical to such a response? - Using a mapping exercise, can you explore how these actors relate to one another? - Do you know any relevant policies that have been developed (or may be in development) for guiding mitigation and preventative responses to climate change? |

| Tips about this session |
| --- |
|  |

| Session 4 Community engagement strategies |
| --- |
| Objectives:   - To identify existing community engagement strategies, rank them and explain why they are important |
| Considerations for different sites:   - Keep the same breakout groups as before - Flexibility here - each site can do break out groups if they decide |

| Expected outputs | Checked (yes/no) |
| --- | --- |
| List of CEI strategies used for climate change events |  |
| Explanation of why these CEI strategies is important |  |

| Supporting Materials | Checked (yes/no) |
| --- | --- |
| List of CEI strategies |  |
| Reporting template |  |

| Instructions for exercise |
| --- |
|  |
|  |

| Tips about this session |
| --- |
|  |

| Session 5 Study design |
| --- |
| Objectives:   - To get feedback on the initial Theory of Change (ToC) and integrate participants perspective into the study design |
| Considerations for different sites:   - For the same day, keep all groups inclusive - Decide about a core group here and decide when they meet at a later stage - Lighten up session - keep some points only for core stakeholder group - Flexibility from sites - about pushing session 4 before lunch- putting lunch at 15:00 or have lunch and offer |

| Expected outputs | Checked (yes/no) |
| --- | --- |
| Notes on Theory of Change and suggestions for way for forward |  |

| Supporting Materials | Checked (yes/no) |
| --- | --- |
| Theory of Change Figure 5 |  |

| Instructions for exercise |
| --- |
|  |
| Present Theory of Change (ToC) used in initial proposal  Gather input of ToC through interactive discussion |

| Tips about this session |
| --- |
|  |

| Session 6 Concluding remarks |
| --- |
| Objectives:   - To deliver thank you note and appreciation to participants - To provide a quick summary of the workshop - To state action points and way forward |
| Considerations for different sites:   - Keep in mind different finishing times per site |

| Expected outputs | Checked (yes/no) |
| --- | --- |
| List of stakeholder names for core group |  |

| Supporting Materials | Checked (yes/no) |
| --- | --- |
|  |  |

| Instructions for exercise |
| --- |
|  |

| Tips about this session |
| --- |
|  |

# SECTION IV: GENERAL FACILITATION SKILLS

**Facilitators skills document**

**Content**

1. Understand your role as facilitator:
2. Before the event
3. Beginning of the event
4. While presenting
5. While moderating
6. Towards the end of the event
7. Recommend videos

**Document**

1. ***Understand your role as facilitator:***

- First, you're helping people move towards specific goals or outcomes. There's some states they want to reach at the end of the workshop that they don't have at the beginning.

- Second, you're creating and leading a group process that moves them forward together. This means you can leverage opportunities for interaction and collaboration that will help people shift in a way that they might not be able to independently.

- Third, you're creating an environment where each individual person can actively participate in and contribute to the process. This requires you to move from being just a good presenter to someone who can bring out the best in others.

1. ***Before the event***

- Understand the agenda for the consultation. Repeat agenda and solve any prior issues that might arise
- Have a team meeting one day prior to the event to organise yourself. Motivate yourself and the team, remember this work fits into a broader agenda for GDAR but also for our planetary health and wellbeing.


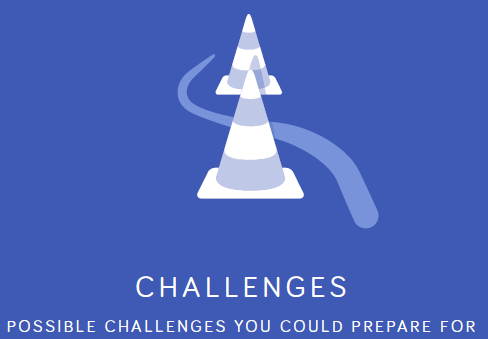


- The follow list will help you to clarify aspects:
- In what order will you present topics?
- How will participants get to know each other (icebreaker)?
- How will they understand the objectives? Find techniques that are useful for prioritizing issues to reach a consensus.
- Will all participants be in every session?
- How and when will break-out groups feedback to the wider group?
- When will you recap and summarize?
- How will the outcomes of one session flow into the next?
- How to make sure documentation and discussion are being collected?
- How will you achieve closure of the overall events?
- Prepare yourself for possible challenges during the day. Listing down possible challenges and solutions would be useful.
- For example: Have a “Plan B”, in case the internet is not working.

1. ***Beginning of the event***


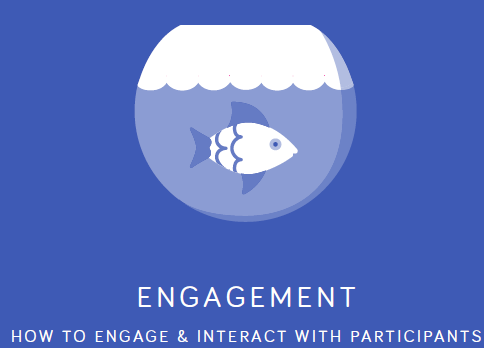


- Welcome participants warmly.
- Answer as much as possible all their question
- Be respectful of all participants and attendees
- Before speaking, introduce yourself to the Group (and the public).


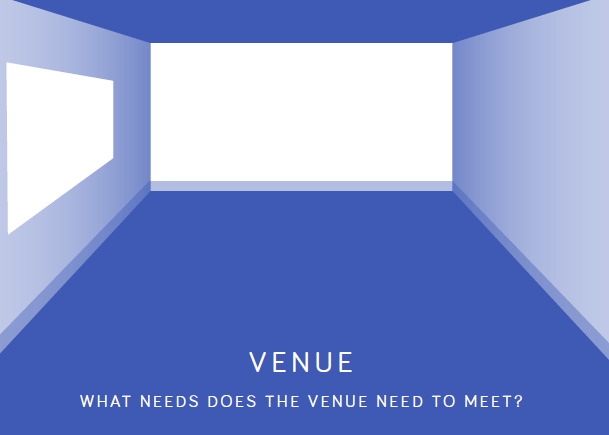


- Give some time to participants to accommodate themselves in the room.
- Have some time to explain where facilities are (toilets food, emergency exit)
- Introduce the team: Introduce what are the team responsibilities so they can refer accordingly in case they have any question or need support.
- Identify the possible distractions that you might run into in the overall workshop and try to address them from the beginning.

1. ***While presenting***

*Notice, this sessions might bring lots of questions from participants

- Listen carefully first and camly, direct the question to the person in the room that can answer it properly (your PI for example).
- One person speaks at a time, as recognized by the Chair.

Familiarise yourself with:

- The presentation
- Terms
- And do a quick research about different example and case
- Presentation skills
- Speech and delivery
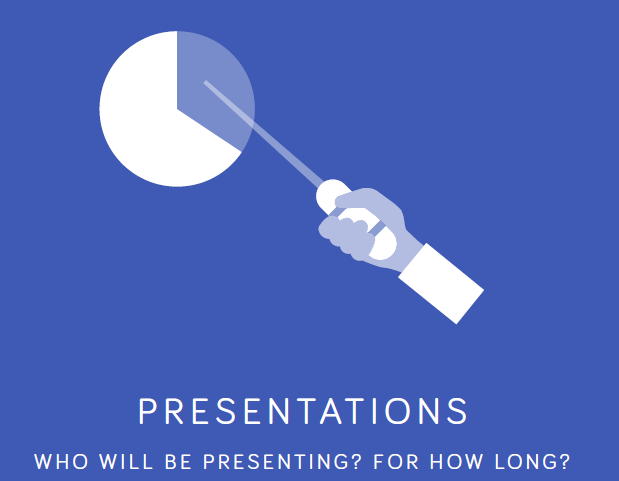

- Speed
- Using shared screens
- Knowledge translation skills
- From science to lay
- Simplifying the terms
- Presenting academic findings in simple

*The scientific content might be very complex to understand. Try to make it light, comprehensive, and you could use one local reference to describe a concept.

- Use an include language, avoid making personal comments during the ice-breaker exercise
- Use a motivation and encouraging language

1. ***While moderating***

- **Build Rapport**- smile, make eye contact, and allow introductions; create a warm, supportive, and comfortable environment.
- **Be an Active Listener**- focus on what is being said; use respondent comments as you paraphrase/summarize; nod your head; lean forward as you listen.
- **Remain Neutral yet Involved**- maintain objectivity both verbally and non-verbally; remember 80:20 rule- the participants talk 80% of the time and the moderator 20%.
- **Be Flexible**- adapt to the flow of the discussion; remain open to changes in the moderator’s guide; adjust to client’s requests during the group; change your physical behavior-sit, stand, or walk around the room.
- **Use the “5-second Pause” and “Probe” Techniques**- ask clear questions and pause for consumers’ responses; probe for more information/ clarity of comments- avoid asking why.
- **Acknowledge and Respect**- recognize each participant throughout the focus group session; respect various points of view, and emphasize respect among the group.
- **Practice good Organization/Management Skills**- practice the guide, prepare for the unexpected; keep the discussion moving, focused, and within the established timeframe.
- **Have Knowledge of the Topic**- basic information on the subject helps in probing areas for more in-depth discussion; demonstrate a degree of naïveté.
- **Be Enthusiastic and Attentive**- have a high energy level; pay attention to participants- recognize group dynamics.
- **Have a Sense of Humor**- laughter keeps the group relaxed, encourages sharing of information, and helps the moderator maintain a human connection.

***6) Towards the end of the event***

* Notice: Closing the workshop is highly Important is almost more important than how you start it.

*With participants*

- Do a quick review of the material we covered.
- Give people time to synthesise their own key takeaways.
- Have your participants create an action plan. If you're asking them to do any post event work or extended learning, you can present that first, but give people a handout where they can write down their action plan.
- Leave the group on a high note of inspiration.
  - For example, with a fun video, or great quote to share at the end/
  - Ask people to close by sharing something their feeling or that they learned.
  - If you have time, it's nice to go around the room, and hear from everyone. Just tell them two to three words, so it's brief. B

*With your team*

- Make sure to congratulate them
- Make sure you have all the outputs needed
- Make sure you have all the next steps and action point list
- Take time to reflect in what came up positively

Suggested videos:

VIDEO 1 - [How To Be A Great Facilitator - The 8 Facilitation Skills You Need](https://www.youtube.com/watch?v=5kPP07jY_rQ)

VIDEO 2 - [3 icebreakers that work anywhere, anytime ⛸](https://www.youtube.com/watch?v=JfT8wipIV1I)

**Supplementary File 4: Stakeholder Report**

Global Diet and Activity Research Network (GDAR)

Stakeholder Consultation Workshop No.1

**(Brazil, Observatory for Urban Health in Belo Horizonte, Federal University of Minas Gerais OSUBH-UFMG)**


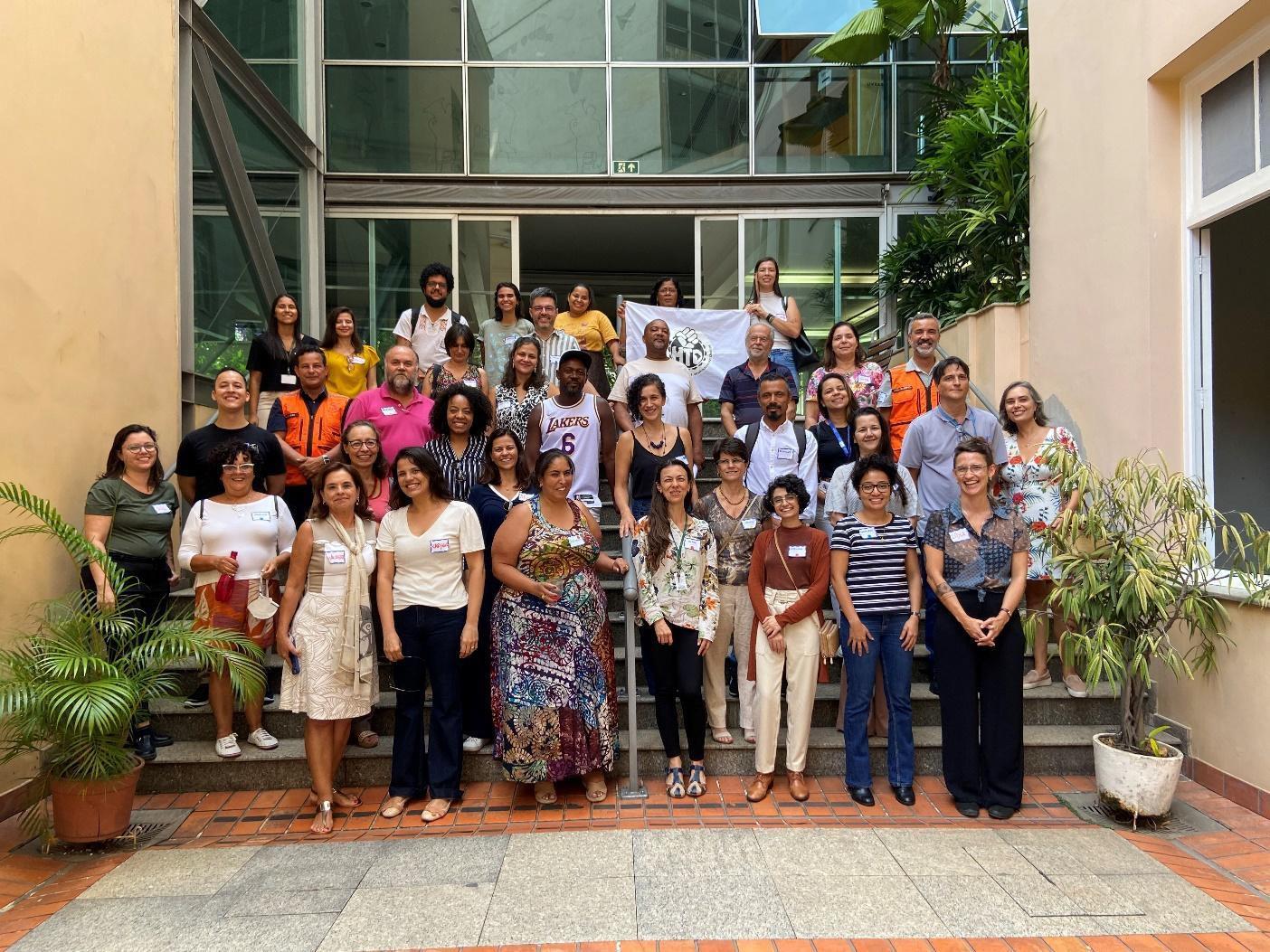


**Brazilian OSUBH-GDAR team responsible:** Lidia Maria de Oliveira Morais, Larissa Lopes Lima, Adalberto Lopes, Amanda Magalhães, Elis Borde, Waleska Teixeira Caiaffa

**Brazilian local OSUBH team responsible:** Debora Coelho, Solimar Rocha, Uriel Moreira Silva, Karynna Ferreira, Magda do Carmo Parajára

### **Observers:**, Amélia Augusta de Lima Friche, Aline Dayrell, Luana Lara Rocha

**15th March 2023**

**Contents**

1. [Introduction 3](#_heading=h.cisc1j5ofsxh)
2. [Overview of GDAR and workshop objectives 3](#_heading=h.4l7yyyv7d1pc)

[GDAR’s aims](#_heading=h.ugmaljye1fwk) 3

[Workshop objectives 3](#_heading=h.esp47xd7ukay)

1. [Syndemic variables 3](#_heading=h.pwurfcs1x2xl)
2. [Top climate change events 6](#_heading=h.qnii9yai94b5)
3. [Local policy, commercial, and community actors 8](#_heading=h.2l5ux2cct7k)
4. [Community Engagement Strategies 10](#_heading=h.qn3flkq6atd9)
5. [Theory of Change 11](#_heading=h.dlefq3t8d7dm)
6. [Who should also be present next time? 12](#_heading=h.7e15atb071nm)
7. [What’s next?](#_heading=h.y1fwpkggjrlo) 12
8. [Acknowledgment 12](#_heading=h.x3virgcbswfq)

# Introduction

The Global Diet and Activity Research (GDAR) Network addresses the rising non-communicable diseases (NCD) burden in low- and middle-income countries (LMICs). It focuses on built and food environment determinants of diet and physical activity (PA). Its "GDARSpaces (2021-2025)" project proposes a syndemic approach to address built and food environment vulnerabilities to support and enhance healthy diets and physical activity.

As a significant activity of GDARSpaces, the Belo Horizonte team organised its first multi-sectoral stakeholder consultation, "Risco e resiliência frente às mudanças climáticas: Mapeando experiências em Belo Horizonte" or *Risk and resilience in the face of climate change: Mapping experiences in Belo Horizonte.* The Belo Horizonte GDAR team held the first consultation on Wednesday, March 15th, 2023.

**Venue:** Conservatório de Música da Universidade Federal de Minas Gerais, Belo Horizonte, Brazil.

**Attendance:** 26 stakeholders (policymakers, NGOs, advocacy groups, local government representatives, academia, technical specialists, civil society members); 3 observers; 11 team members (6 directly linked to GDAR).

# Overview of GDAR and workshop objectives

The GDAR Network aims to contribute to decision-making for healthy societies by:

## GDAR’s aims:

- - Develop interventions responding to urbanisation, climate change, and socio-environmental vulnerability.
  - Explore how commercial, policy, and community actors and actions influence food and built environments and diet and PA behaviour in local contexts.

## Workshop objectives

- - Identify stakeholder priorities.
  - Identify priority urbanisation and climate change hazards.
  - Identify key actors in syndemic risks and response.
  - Co-design community engagement and involvement strategy and pathways to avoid the health impact of climate change.

# Syndemic variables

One of the objectives of GDAR Spaces is to conduct environmental audits of different syndemic variables that contribute to unhealthy diets, physical inactivity, and health risks. "Syndemic" refers to those factors or diseases that can further interact, resulting in worse health outcomes than each factor or disease would likely have generated.

*GDAR team gave presentations on:*

- - *Syndemic approach: different environmental and climatic variables impacting the food and built environment and health.*
  - *Urbanisation and climate change impact food and physical activity behaviours, affecting the risk of non-communicable diseases.*
  - *The link between urban design and sustainability, discussing how environmental characteristics may be linked to physical activity in the context of Belo Horizonte city.*
  - *The complexity of the urban environment.*

The participants interacted with the presentation, answering the question proposed:
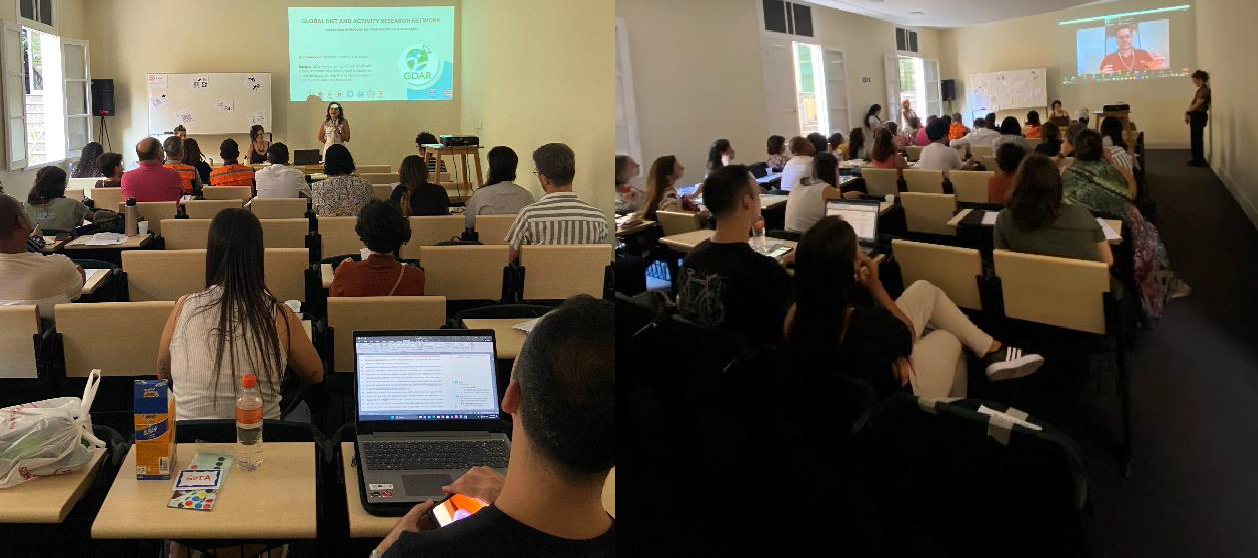


“What other indicators do you consider relevant and available for assessing Belo Horizonte urban environment?”

In the plenary, they listed the variables they felt could be incorporated and suggested some data sources and possibly interesting tools. At the same time, one of our team members filled the board with the keywords, and assembled close to where guiding icons had been previously placed.


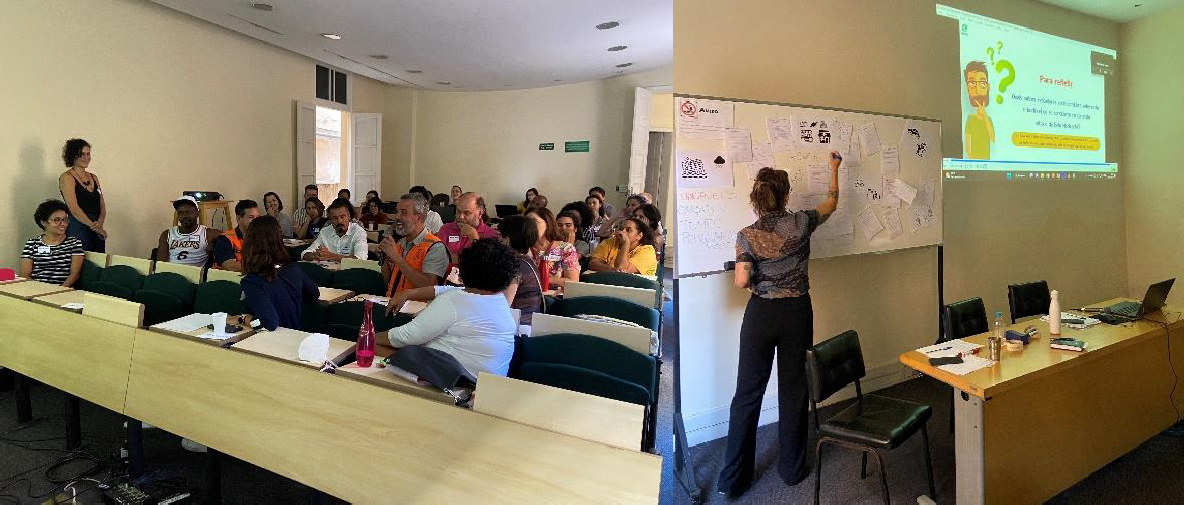


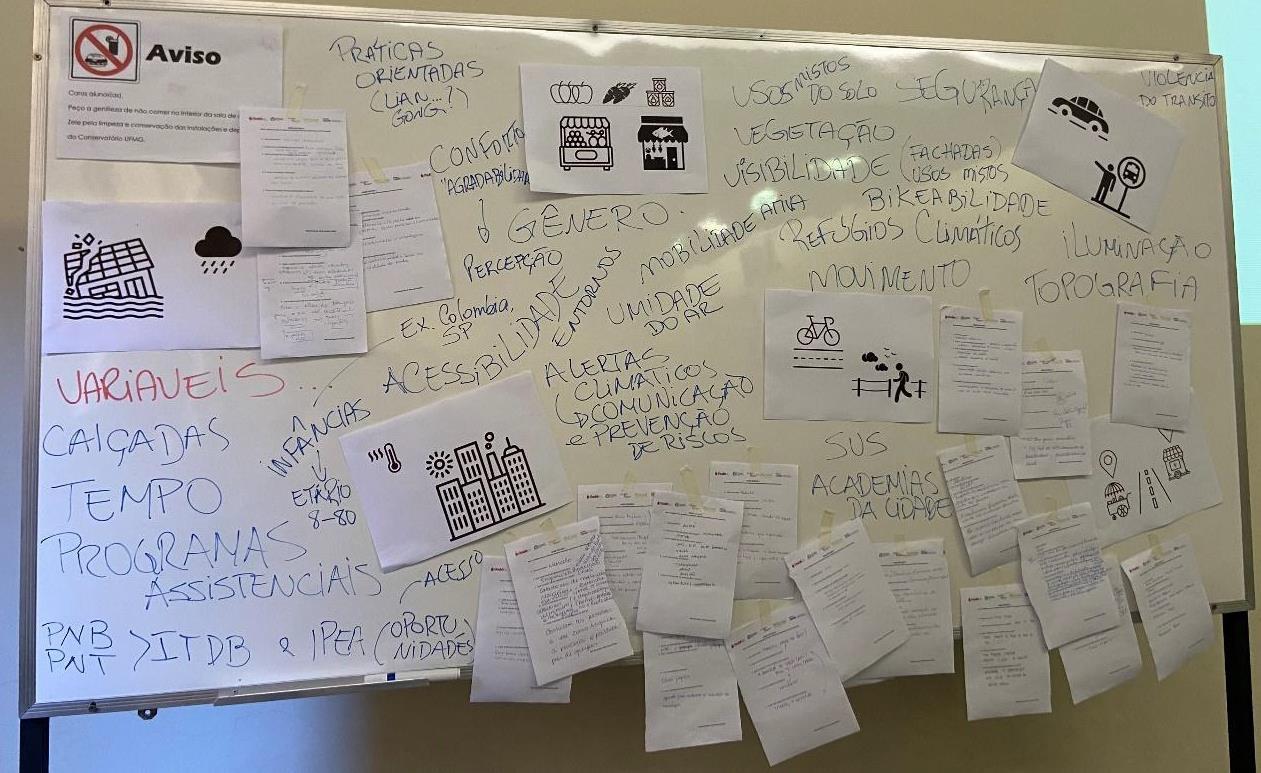


Variables listed by the participants:
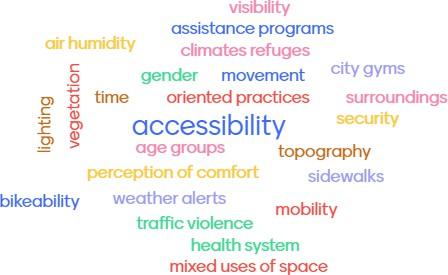


Other data sources, studies, and tools were suggested:

- - ‘Map of Opportunities’ - Available at <<https://www.ipea.gov.br/acessooportunidades/mapa/>>.
  - ‘Al colegio en bici’, initiative in Bogotá.
  - See NETWORK/CARTOGRAPHY/CONSTELLATION for the organization of social actors: research, professional performance, nurseries of citizen initiatives, 'Update Politics' (<https://www.institutoupdate.org.br/>), 'Personal Brain' (<https://www.thebrain.com/>), for a summary of indicators. Examples:
    - <https://www.institutoupdate.org.br/sobre/>
    - [https://emergenciapolitica.org/america-latina](https://emergenciapolitica.org/america-latina/)
    - Vivero de Iniciativas Ciudadanas: <https://vicvivero.net/> and [https://www.civics.cc/pt/#!/iniciativas](https://www.civics.cc/pt/%23!/iniciativas)

Remarks on the plenary discussion:

- Interaction between the urbanization process and climate change intensifying the vulnerabilities of the urban environment.
- Urban contexts that promote or do not healthy eating and physical activity.
- Causes of causes of NCDs.
- Differentiation between social and built context.
- The variable of time being subject to housework and care work/responsibilities.
- Importance of multisectoral actions.
- Gender in public spaces involving fear of crime, being subject to time of the day/public lighting.
- Maintenance of public sports facilities.
- Mixed use of land / Accessibility of services by walking distance relieves the need for motorized transportation.
- Promotion of healthy behaviours (active mobility, cycling and walking, quality and comfort of the streets by people's perception, traffic violence).
- Culture of prevention – institutional communication regarding localized rainfall.
- Climate change adaptation initiative led by the Municipality: “Rain Gardens”.
- Vegetation – shade – pleasant environments for pedestrian.

The session was broad and rich in content. The participants demonstrated interest and were willing to participate in the debates. After the variables discussion session, the workshop moved into the next session, which focused on climate events.

# Top climate change events

Climate change refers to long-term changes in temperatures and weather patterns. In GDAR Spaces, climate change events are used as a lens through which policies and interventions related to health can be examined. The project aims to understand how policy and community initiatives are triggered as a response to specific climate events. The objective of the session was to invite participants to identify relevant climate change events that have occurred in the last 10 years in Belo Horizonte City and their effects on health.

A short introduction on the theme of types of climate change events with a few examples was delivered, and the participants were split into five groups of about five people.

First, the groups discussed and listed relevant climate change events and their effects on health for ranking them by order of importance. To each group, one facilitator from our team was assigned to help orient the discussion and solve any questions. Each group decided who would be the participant to report back to the plenary. A template was offered to guide the conversation and facilitate registering it.


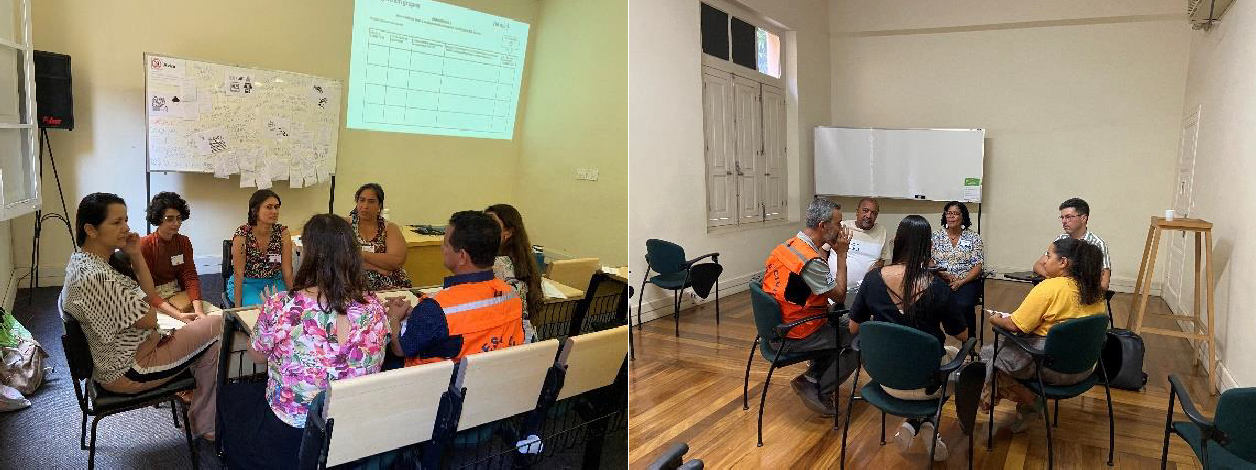


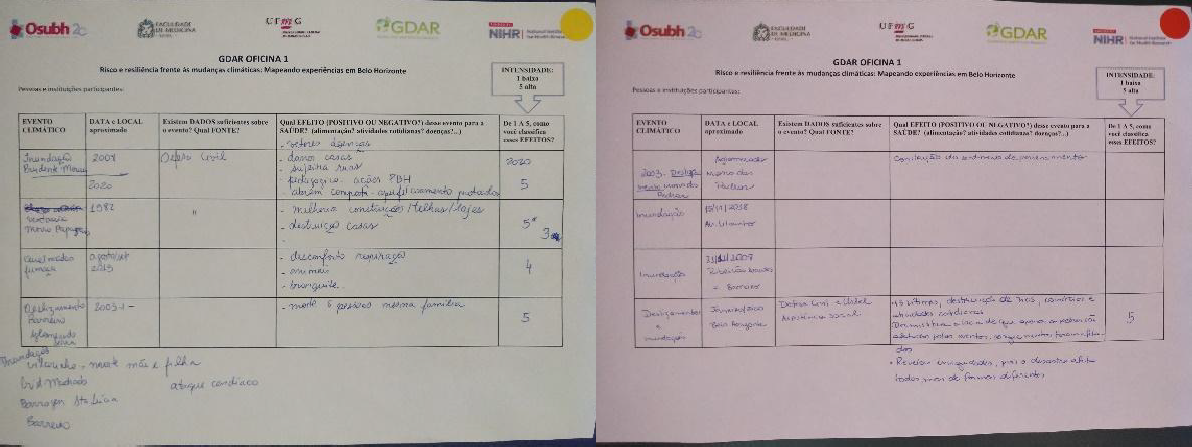


The most impactful event listed was the heavy rainfalls in 2020 and their consequences, such as floods and landslides. Other serious rainfall events were recorded for 2018, 2014/2015, 2008, and 2003, with

similar impacting effects, often in the same neighbourhoods and areas of the city. Some of the degrading effects listed were:

- Deaths due to floods and landslides.
- Destruction of roads, businesses, and daily activities.
- Psychosocial illnesses, desolation, sadness.
- Reduced mobility.
- Increase in cases of waterborne diseases, leptospirosis, and vector disease such as dengue.
- Impact on agriculture, with a reduction in the quantity and quality of food and, consequently, an increase in prices, impacting municipal and family budgets.
- Pollution (water and air) consequences.
- Insecurity of the population that remained living near the flooding sites and of the people who routinely travel on these roads.
- Most vulnerable surrounding areas are the most affected.

One participant from the Civil Defense reported that during the 2003 heavy rainfall event, an entire family died (11 people). The background is that this family had been removed from Morro das Pedras (a slum area in Belo Horizonte) due to the risk of landslides but returned there a few days before the disaster. This report highlighted the need to work directly with the population, to raise awareness of the risks and also on public policies to find better solutions for people living in risk areas. It leads to the fact that despite all the adverse effects, some positive unfoldings of the disasters were listed:

- Start a discussion on the benefits and harms of plugging rivers.
- Assembling of the ‘Grupo Executivo de Áreas de Risco’ (GEAR).
- Motivation for urbanization projects for villages and slums.
- Belo Horizonte municipality actions: pedagogical, preventive monitoring.
- Improving the performance of Civil Defense, incentives, and plans.
- Perfecting protocols for emergencies (for example: opening floodgates).
- It revealed inequities, as the disaster affects everyone but in different ways.
- Building a sense of belonging in some affected areas.
- Create a “prevention culture” among policymakers and the community.

Also, wildfires in the city surrounding green areas were listed in 2019/2020, leading to the destruction of natural habitats, smoke, and soot in the air, which decreased air quality, generating breathing discomfort and increased cardiovascular and respiratory diseases risk. Last but not least, the unprecedented collapse of mining tailings dams, linked to the volume of waterfalls in 2019, contaminated the river basins around the city and implicated in water shortage and mental degrading effects, among others, that were out of the scope of this report.

See more on the Climate Events synthetic table attached.

# Local policy, commercial, and community actors

Second, the groups chose one of the climate change events they listed and described what coping strategies were ongoing or planned to address the effects of the event. An important component and objective of GDAR Spaces is to understand better how community, policy, and commercial actors (and their respective institutions/groups) respond, cope, and adapt to climate change shocks.

Accordingly, the groups have a rich list of social actors involved in different levels and roles to cope with the listed climate change events, their relationship, and related public policies.

A quick stop for coffee break and networking helped freshen up the ideas.


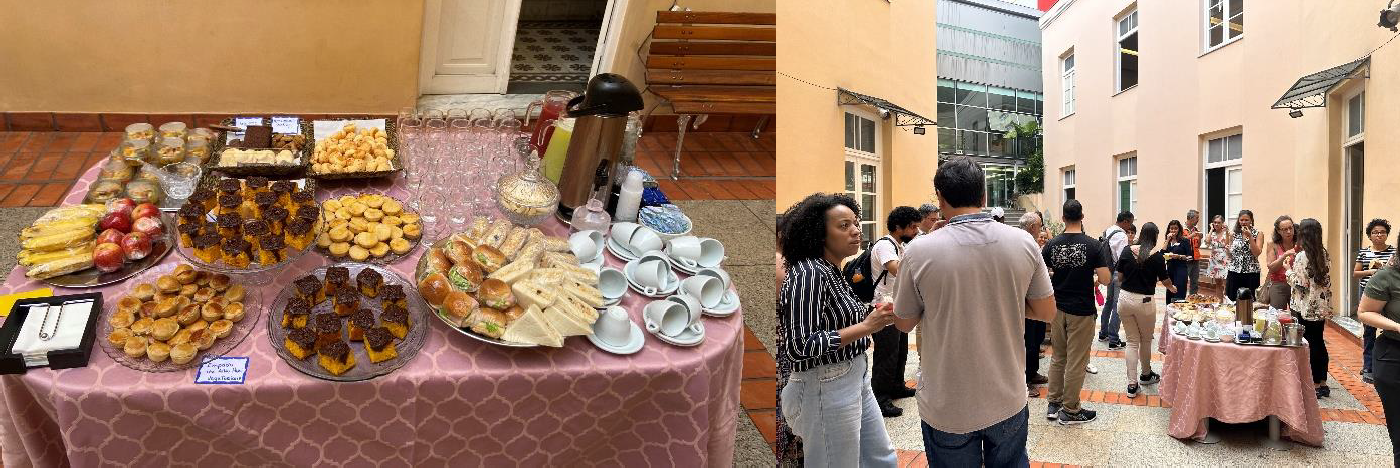


Back on plenary, a representative of each of the five breakout groups provided feedback on their list of climate change events, and the top ones chosen to be analysed in depth by all groups were the heavy rainfalls and related effects, such as floods and landslides, as reported on the Climate Events synthetic table.


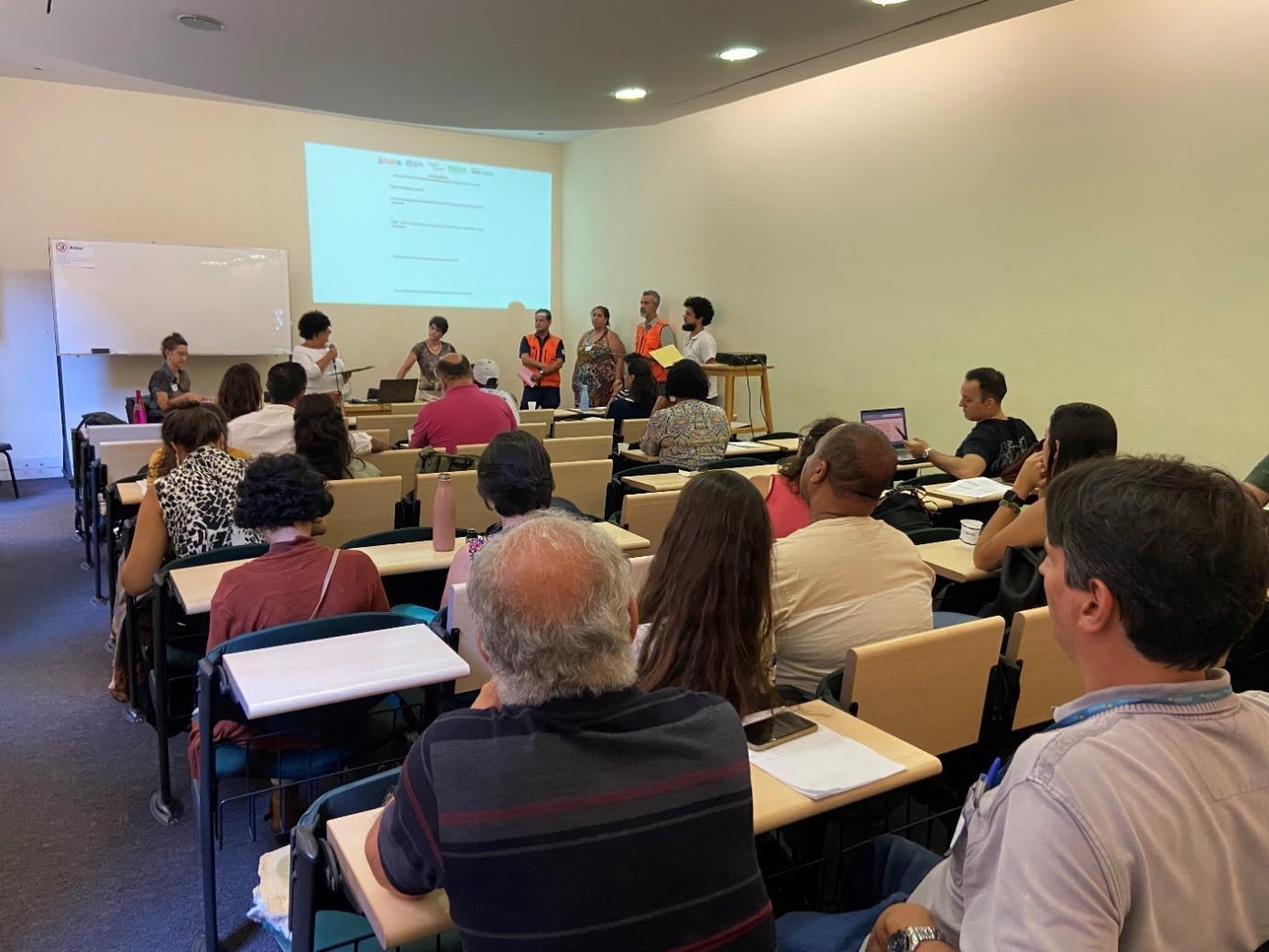


To that chosen event, the participants listed strategies to cope with the climate event, the key actors involved in resilience strategies, and the relevant policies that have been developed (or maybe are in development) for guiding mitigation and preventative responses to this and other similar climate change events in Belo Horizonte.

After the participants’ presentation, the observers, who also participated in the group work helping moderate and orienting notetaking, also shared their observations and insights during the discussions.

Resilience strategies:

- Urban planning education in schools by the municipality.
- Organized civil society proposed “10 PROPOSALS TO DEAL WITH FLOODS IN BH: How to make the waters generate well-being instead of tragedies?” (Available at:

[<https://manuelzao.ufmg.br/wp-content/uploads/2020/02/10-propostas-texto-final-](https://manuelzao.ufmg.br/wp-content/uploads/2020/02/10-propostas-texto-final-)web.pdf>).

- Infrastructure works and mobilization of resources for adaptation and retention networks.
- Greater communication in risk areas, alerts of heavy rains and possible flooding locations for the general population.
- Municipal “Rain gardens”.
- Waste management space in order to reduce the obstruction of water channelling routes and the silting up of rivers.
- Urban gardening/food production areas incentives aiming to increase soil permeability in vulnerable regions.
- Risk Area Programs (Civil Defense and Risk Area Management).
- “Piscinão” (containment basins).
- Infrastructure containment works of slopes in risk areas.

Actors involved in these strategies encompassed civil society, City Hall bodies, inspection institutes, participatory government instances, university/academies, social movements, community associations, organized civil society, Risk and Disaster Management Group, social assistance bodies, and communication (various media). One participant shared a comment that highlights the perception of inequities and the effects of climate change events.

“For the rich, resilience is accompanied by economic power, resilience for the poor resilience comes from the shared experience of the event and from community support.”

Related public policies were broad and cross-sectional and will be the subject of further analysis. Find more on the Climate Events in the table attached.

# Community Engagement Strategies

Community Engagement and Involvement (CEI) is a core component of GDAR Spaces research. To undertake the research in collaboration with the communities most likely affected by the research outcomes.

As part of the closing session, in plenary, participants were asked to brainstorm on how to continue with community engagement activities they would desire to participate in and support, looking forward to future engagement in GDAR Spaces.


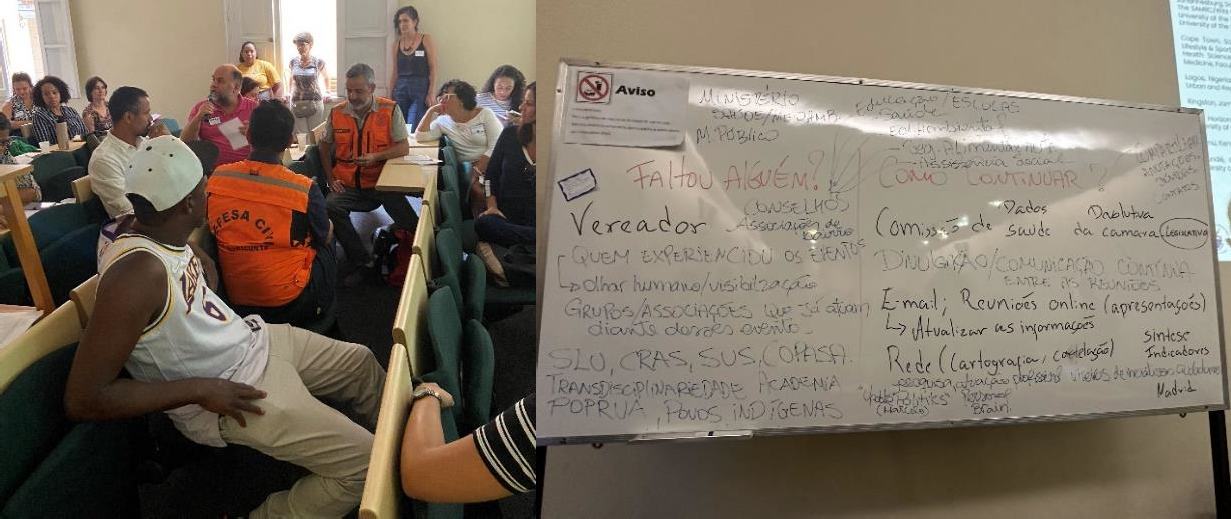


The highlights of the discussion were:

- - Articulation with the Health Commission of the Legislative instances.
  - Disclosure of information and ongoing communication between meetings.
  - An email with periodic updates on meeting topics and escalations.
  - Online presentations on specific topics.
  - Share data results and the GDAR-OSUBH project's next steps.

# Theory of Change

The theory of change (ToC) of GDAR Spaces maps out how changes are expected during the research. The ToC identifies inputs, activities, outputs, outcomes, and impacts that drive the study and the critical underlying assumptions that need to be met to bring about change.

The idea of the Theory of Change was explained to the participants in the introductory part of the meeting in a simplified manner, and mentioned throughout the different steps, indicating its guiding principles in our activities, and incorporating the workshop outputs in our subsequent activities.


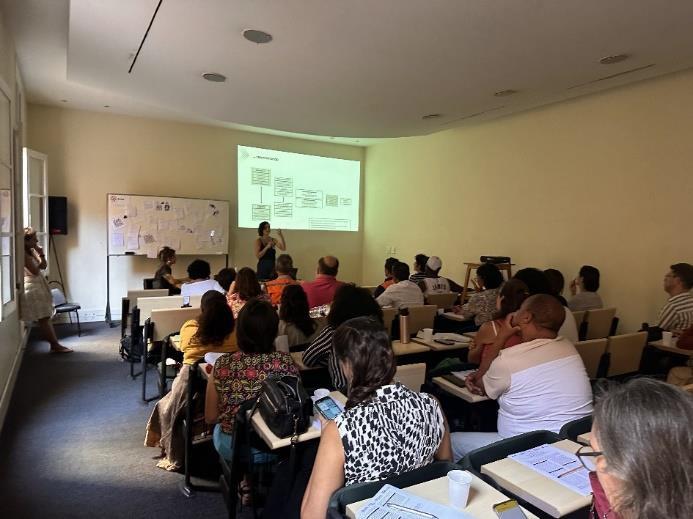


# Who should also be present next time?

Also, in plenary, they were asked to list what other social actors should have been at the workshop or should be invited to next meetings for their unique perspectives and/or experience. The list was the following:

- - Ministry of Health.
  - Ministry of the Environment.
  - Republic's General Attorney Office (originally *Ministério Público*)
  - Municipal and state boards/commission on: Education, Health, Environment, Food and Nutritional Security, Social Assistance (originally *conselhos municipais e estaduais*)
  - Neighbourhood associations.
  - Municipal legislative power representative.
  - People who experienced the events (incorporating sensitivity and promoting visibility).
  - Groups and associations that work with extreme weather events/disasters.
  - Urban Cleaning Service (SLU).
  - Social Assistance Reference Center - CRAS/CREAS.
  - Sanitation Company of Minas Gerais – COPASA.
  - Street population.
  - Indigenous peoples.

# What’s next?

This workshop proved to be an insightful opportunity for both the researchers and the participants. Observers also had an insightful perception of the process. Some comments received during the event highlight that:

- “The workshop was powerful in the sense of bringing together different visions and boosting public policies”.
- “Updating what you already knew and getting to know new practices”.
- “I will take the discussion home and to my work environment”.

The transdisciplinarity and intersectoral discussions demonstrated the interest in the subject and possible ways for co-creating research and action on climate change in Belo Horizonte, based on what is already happening and all the assets available.

It was agreed that the GDAR-OSUBH team would keep in touch with participants, make an effort to include those absent but who could have contributed, and invite them to the project’s next steps.

Next GDAR workshop on Systems thinking: possibly July 2023.

Next GDAR workshop following up to this first one: December 2023.

# Acknowledgment

Many thanks to all participants, OSUBH team who supported all the logistics, and observers, who kindly supported group work moderating and notetaking and sharing their insights at the final plenary. The GDAR project is funded by the NIHR (NIHR133205) through UK aid from the UK Government to support global health research. The views expressed in this publication are those of the authors and not necessarily those of the NIHR or the UK Department of Health and Social Care.

# Supplementary File 5. Validation of Syndemic Variables Across Domains

Legend: Green = strong consensus of high relevance; Yellow = Split validation of high/low relevance; Red = strong consensus of low relevance.

| Domain | Variable | Consensus Level | Notes |
| --- | --- | --- | --- |
| Climate & Natural Hazards | Air pollution (PM₂.₅) | Green | Validated as highly relevant across all sites; major health determinant |
| Climate & Natural Hazards | Air pollution (PM₁₀) | Green | Strong relevance to NCD outcomes |
| Climate & Natural Hazards | Flooding | Green | Varied by geography and infrastructure resilience |
| Climate & Natural Hazards | Precipitation | Amber | Varied by geography and infrastructure resilience |
| Climate & Natural Hazards | Urban Heat Island | Green | Strong relevance to NCD outcomes |
| Built Environment | Walkability | Green | High consensus; supports active mobility and health |
| Built Environment | Sidewalk presence | Green | Validated as crucial for accessibility |
| Built Environment | Sidewalk obstruction | Red | High consensus of low relevance |
| Built Environment | Public transport points | Red | High consensus of low relevance; less critical in some cities |
| Built Environment | Road Safety | Amber | Varied by geography, linked to safety perceptions |
| Built Environment | Number of shops | Red | High consensus of low relevance; less critical in some cities |
| Built Environment | Urban density | Green | Strong relevance to NCD outcomes |
| Built Environment | No. of public spaces | Amber | High consensus of low relevance |
| Built Environment | No outdoor physical activity spaces | Green | Strong relevance to NCD outcomes |
| Built Environment | Garbage presence | Green | Strong relevance to NCD outcomes |
| Built Environment | Streetlights | Amber | Context-dependent; linked to safety perceptions |
| Built Environment | Green space presence | Amber | Split view of relevance |
| Food Environment | Healthy food outlets | Green | Strong relevance validated across sites as key determinant |
| Food Environment | Unhealthy food outlets | Green | Strong relevance validated across sites as key determinant |
| Food Environment | No of food retail | Amber | Mixed validation; varied by city density |
| Food Environment | Healthy food’s shelf length | Amber | Split view of relevance |
| Food Environment | Informal food location | Amber | Split view of relevance |
| Food Environment | Formal food location | Red | Low relevance |
| Food environment | Food advertisement presence | Green | Strong relevance to NCD outcomes |

# Supplementary File 6: Additional Syndemic Variables Proposed by Stakeholders

| Domain | Variables |
| --- | --- |
| Climatic | Environmental degradation: oil spillage, water contamination, waste management, water wastage, power lighting instead of natural lighting, chemical spills into the drainage system, dumping, land degradation, freshwater depletion;  Weather events: heavy rain, drought, strong winds, heat days, seasonal rain changes, wildfires, urban fires, sea level rise, weather alerts, climate refugees, air humidity;  Ecological and socio-cultural: impact on animals and increase in disease, biodiversity loss and appearance of new species, disruption of the ecological balance, sociocultural factors, migration. |
| Physical Activity | Infrastructure: number of gyms, presence of recreational activity; Safety: number of first aid at physical activity sites, personal safety, vandalism of public community spaces, traffic violence, visibility;  Urban planning: non-respect of the urbanization plan, non-involvement of resource persons in community development decision-making, accessibility, vegetation, topography. |
| Food Environment | Production, processing, and distribution: food production chain, food supply chain, food manufacturing and industry, food transportation and storage, food logistics, food handling, agricultural practices, agriculture industry, variety of agriculture, small-scale farmers, use of chemical fertilizers, use of pesticides, use of insecticides, wholesale, food security and storage, sustainability and competitiveness of food products, food waste;  Accessibility and safety: food prices, inflation of food products, food quality/cost ratio, expiry dates, food labelling, food safety, health and safety, proportion of household budget spent on food, power outages, assistance programs, health systems;  Cultural and social: meat eating culture, school food environment, corporate foods, community and school vegetable gardens, overregulation, gender, age groups, time, perception of comfort. |

# Supplementary File 7. Detailed Linkages between Climate Events and Health Reported by Participants

This expanded table presents the complete qualitative mapping of climate-related health impacts as described by workshop participants across multiple domains.

| Category | Health Impacts per Category |
| --- | --- |
| Deaths | Deaths due to floods, landslides, house collapsing, heat-related mortality, loss of animal lives (ground and water animals). |
| Mental Health | Psychosocial illnesses, desolation, sadness. Disruption in the quality of life, causing suffering and discomfort. |
| Waterborne Diseases | Waterborne diseases such as leptospirosis and vector-transmitted diseases like dengue, malaria and schistosomiasis. Water shortage with use of contaminated water leading to diarrhoea and dehydration. |
| Food Environment Risk Factors | Food insecurity and malnutrition due to flooded gardens and delayed harvests, raising food prices and reducing sovereignty. Use of polluted water for irrigation, loss of aquatic animals, increased salinity in coastal aquifers, land degradation, and infrastructure damage (shops, storage). Increased risk of foodborne disease and unhealthy diets due to displacement. |
| Pollution | Air contamination from motorised vehicles and waste burning, causing unpleasant smells and respiratory risk. Water contamination reducing safety and access, increased diarrhoeal disease, high salt in freshwater ecosystems, wildfire smoke reducing air quality and outdoor activity. |
| NCD-Related Risk Factors | Dehydration, exhaustion, respiratory problems, headaches, heat stroke, waste-burn emissions linked to cancer, and general deterioration of physical health. |
| Physical Activity | Inaccessibility of PA spaces due to damage, reduced outdoor activity because of heat and pollution, reduced PA in schools and sports due to water shortages. |
| Other | Poor indoor ventilation, increased hospital pressure, reduced sanitation, and disrupted breastfeeding routines. |

# Supplementary File 8. Multi-sectoral Actors Engaged Across Study Sites

This table maps multi-sectoral actors identified across seven urban study sites, illustrating the institutional diversity shaping local responses to climate–health risks.

| Category | Brazil (Belo Horizonte) | Nigeria (Lagos) | Cameroon (Yaoundé) | Jamaica (Kingston) | South Africa (Cape Town) | South Africa (Johannesburg) | Kenya (Kisumu) |
| --- | --- | --- | --- | --- | --- | --- | --- |
| Government Agencies | City Hall, inspection bodies, participatory gov. instances (e.g. Food Security Council). | Min. of Education, Min. of Sports, LASRO, Min. of Environment, Min. of Works, Local Gov. Affairs, Youth, LAWMA, Lagos Urban Dev. Initiative. | Min. of Education, Environment, Works, Youth, Agriculture, Energy & Water, Local Gov. Affairs. | Min. of Agriculture & Fisheries, Health & Wellness, Industry & Commerce, Economic Growth, NEPA, Kingston & St. Andrew Municipal Corp. | Provincial/National Depts: Education, Health, Transport, Agriculture, Spatial Planning, Parks, Disaster Risk, Social Development. | City of Johannesburg, Depts of Planning, Health, Transport, Energy, Agriculture, Environment, National & Provincial Depts. | County Gov. of Kisumu – Depts of Environment, Urban Planning, Health, Agriculture, Social Services, Trade, Gender, Youth Fund, Treasury. |
| Civil Society & Community Organizations | Civil society, social movements, community associations, risk mgmt. groups, social aid bodies. | CDAs, CDCs, informal recyclers, Heinrich Boll Foundation, NGOs, Community Dev. Associations. | Community associations, informal recyclers, farmers, vendors, Launch Out Foundation, traditional rulers. | Jamaica Environment Trust, Kiwanis, Lions, Social Dev. Commission, Churches, RADA. | NGOs, Open Streets, Neighbourhood Watch, Friends of the Park, Sweetlife, Community Leaders. | Environmental Monitoring Group, Greenpeace, Section 27, Taxi Alliance, Soul City Edutainment Initiative. | Community reps (farmers, retailers, leaders), youth & faith orgs, CBOs (Muungano), Disaster Response Committees. |
| Academic & Research Institutions | Universities. | Health educators, universities. | Universities, health educators. | Universities, Bureau of Standards, Forestry Dept. | Research & academic institutions (ACD, CSIR, RADAR, Medical Research Council). | - | Universities (Great Lakes, Maseno, Jaramogi Oginga Odinga), Research Institutes (KALRO, KEMFRI, KEMRI). |
| Industry & Commercial Actors | - | i-Fitness, telecoms (MTN, Airtel, Globacom), Nestlé, Access Bank, Cadbury, Dufil Foods, Guinness, Coca-Cola. | - | Agro-Investment Corp., JMEA, Petrojam, Water Bottling Cos., Trade Winds. | Commercial fitness, local businesses. | WWF, WITS Global Change Inst., Gauteng City Regional Observatory, CSIR, World Bank. | Safaricom, Equity Bank, Co-operative Bank, Trade & Transport sectors. |
| Environmental & Health-focused Organizations | Environmental & transport NGOs. | Wecyclers, Recycling Point, Shodex Garden, Fish Farmers Assoc., Nigerian Environmental Society, FAO. | FAO, NSPRI, NAFDAC, AirQo, Wecyclers, Heinrich Boll Foundation. | Environmental Foundation of Jamaica, Heart Foundation, Food for the Poor, GEF Small Grants. | - | WWF, Greenpeace, Tiger Brands, Pioneer, McDonalds, Big Oil, MultiChoice, SABC. | NEMA, Kenya Met. Dept, Water Resources Authority, Greenbelt Movement, WWF, Practical Action, HOPE. |
| Media & Communication | University press, community communication channels. | Enviro News. | - | Broadcasting Commission, Met Office. | - | Section 27, Greenpeace, Soul City Edutainment Initiative, Taxi Alliance. | Safaricom, Citizen TV. |
| International & Multilateral Organizations | - | UNOSOP, FAO, UNDP, IPCC. | UNDP, UNEP, NACCIMA, LCCI, NIMASA, FAO. | UNEP, PAHO, UNICEF. | - | World Bank, UN agencies, DSTV, MultiChoice, SABC. | UN-Habitat, UNEP, Red Cross, WHO Kenya. |

# Supplementary File 9. Lessons Learned for Enhancing Transdisciplinary (TD) Research

This table summarises reflections and lessons learned across transdisciplinary research phases, highlighting strategies for strengthening co-learning, stakeholder engagement, and implementation across sites.

| Phase | General Reflection across Sites | Lessons Learned |
| --- | --- | --- |
| Co-Learning Phase | Iterative learning, knowledge generation, and cross-site observation were beneficial. Some sites built on prior workshop experience. Cross-learning was enhanced when stakeholder groups were mixed, though technical terminology required adaptation. Workshops created new spaces for dialogue, yet challenges persisted around representation and limited policymaker participation. | Material Development: Learn from other sites’ experiences.  Language and Terminology: Harmonise terms to foster cross-learning.  Space Creation: Facilitate dialogue to understand mental models and decision processes.  Participant Representation: Send invitations to individuals (not institutions); use flexible agendas to increase policymaker presence. |
| Pre-Development Phase | This phase underscored the importance of clear agendas, dry runs, and innovative engagement activities to bring diverse profiles together. Dry runs proved essential given variable resources across sites. Adapting agendas to different stakeholder profiles was challenging, emphasising local contextual understanding. | Stakeholder Integration: Bridge profiles (e.g., professors, farmers) by identifying shared concerns.  Dry Runs: Validate each site’s resources, logistics, and context before harmonising workshop activities. |
| Mapping Phase | Ensured key actors were present through targeted invitations. Flexible invitations helped some sites improve attendance, while others found individual invitations more effective. Snowball approaches were inconsistent in managing participant selection. | Effective Mapping: Identify relevant stakeholders per phase.  Clear Invitations: Invite named individuals with defined expectations.  Continuous Improvement: Adjust mapping iteratively using participant feedback. |
| Conceptualization Phase | Harmonising diverse knowledge systems and clarifying research objectives were crucial for aligning expectations. Differences in conceptual understanding highlighted the need for clearer communication of research goals. | Harmonisation: Address differing knowledge systems early.  Clear Objectives: Ensure stakeholder understanding of goals and expected outcomes. |
| Capacity Building Phase | Training facilitators was essential for workshop delivery, reporting, and analysis. However, imbalances between thematic expertise and engagement skills limited effectiveness. Time constraints also affected training quality. | Balanced Training: Combine technical and engagement skills in facilitator preparation. |
| Investigation Phase | Communication issues and misaligned expectations revealed gaps between policy, community, and commercial actors. Participants often worked in silos despite shared spaces, underlining the need for clearer operational messages and defined roles. | Mapping and Tools: Refine tools to align expectations and outputs.  Clear Roles: Define responsibilities and expected benefits.  Communication: Create intentional mixed-group sessions to enhance cross-sector dialogue. |
| Implementation Phase | Highlighted the need for trust-building and addressing hierarchical concerns; engagement effectiveness varied by authority level. | Build Trust: Use findings to establish ongoing collaboration.  Address Hierarchies: Promote equitable participation through engagement rules. |
| Reflection and Refinement Phase | Emphasised improved facilitation, preparation, and continuous evaluation to maintain dialogue among stakeholders. Despite participant mix concerns, TD engagement enabled meaningful discourse and actionable follow-up through post-workshop reports. | Improve Preparation: Tailor workshops to site resources.  Active Dialogue: Sustain engagement via smaller meetings and follow-ups.  Continuous Evaluation: Adjust processes based on regular feedback. |
