# Supplementary material for: Urban climate–NCD syndemics in LMICs: a transdisciplinary framework for Health action
Source: Glob Health Action. 2026 Apr 22;19(1):2650971. doi: 10.1080/16549716.2026.2650971 (PMC13104006; doi:10.1080/16549716.2026.2650971)
Supplement: Checklist_Good_Reporting_of_A_Mixed_Methods_Study_GAMMS_d.docx [file ZGHA_A_2650971_SM7266.docx]

Good Reporting of A Mixed Methods Study- GAMMS

| **Items** | **Guide question/description** | **Location** |
| --- | --- | --- |
| 1 | Describe the justification for using a mixed methods approach to the research question | Study design in the Methods sections. Pg 7 |
| 2 | Describe the design in terms of the purpose, priority and sequence of methods | Methods section. Pg 6-12 |
| 3 | Describe each method in terms of sampling, data collection and analysis | Methods section Pg 6-12 |
| 4 | Describe where integration has occurred, how it has occurred and who has participated in it | Methods section . Pg 6-12 |
| 5 | Describe any limitation of one method associated with the present of the other method | Strengths and limitations Pg 43 |
| 6 | Describe any insights gained from mixing or integrating methods | Discussion and Implications for Policy and Practice Pg 36-42 |

The GRAMMS Checklist is from O'Cathain A, Murphy E, Nicholl J. The quality of mixed methods

studies in health services research. Journal of Health Services Research & Policy. 2008;13(2):92-8.

doi: 10.1258/jhsrp.2007.007074.
